# Identification of epigenetically regulated genes involved in plant-virus interaction and their role in virus-triggered induced resistance

**DOI:** 10.1186/s12870-024-04866-3

**Published:** 2024-03-05

**Authors:** Régis L. Corrêa, Denis Kutnjak, Silvia Ambrós, Mónica Bustos, Santiago F. Elena

**Affiliations:** 1 Institute for Integrative Systems Biology (I2SysBio), Consejo Superior de Investigaciones Cientificas (CSIC) - Universitat de València (UV), Paterna, Valencia, 46980 Spain; 2 Department of Genetics, Federal University of Rio de Janeiro (UFRJ), Rio de Janeiro, 21941-590 Brazil; 3 Department of Biotechnology and Systems Biology, National Institute of Biology, Ljubljana, 1000 Slovenia; 4 The Santa Fe Institute, Santa Fe, NM 87501 USA

**Keywords:** Biotic stress, Defense priming, Epigenetics, Histone modifications, Induced resistance, Potyvirus, RNA-directed DNA methylation

## Abstract

**Background:**

Plant responses to a wide range of stresses are known to be regulated by epigenetic mechanisms. Pathogen-related investigations, particularly against RNA viruses, are however scarce. It has been demonstrated that *Arabidopsis thaliana* plants defective in some members of the RNA-directed DNA methylation (RdDM) or histone modification pathways presented differential susceptibility to the turnip mosaic virus. In order to identify genes directly targeted by the RdDM-related RNA Polymerase V (POLV) complex and the histone demethylase protein JUMONJI14 (JMJ14) during infection, the transcriptomes of infected mutant and control plants were obtained and integrated with available chromatin occupancy data for various epigenetic proteins and marks.

**Results:**

A comprehensive list of virus-responsive gene candidates to be regulated by the two proteins was obtained. Twelve genes were selected for further characterization, confirming their dynamic regulation during the course of infection. Several epigenetic marks on their promoter sequences were found using *in silico* data, raising confidence that the identified genes are actually regulated by epigenetic mechanisms. The altered expression of six of these genes in mutants of the methyltransferase gene *CURLY LEAF* and the histone deacetylase gene *HISTONE DEACETYLASE 19* suggests that some virus-responsive genes may be regulated by multiple coordinated epigenetic complexes. A temporally separated multiple plant virus infection experiment in which plants were transiently infected with one virus and then infected by a second one was designed to investigate the possible roles of the identified POLV- and JMJ14-regulated genes in wild-type (WT) plants. Plants that had previously been stimulated with viruses were found to be more resistant to subsequent virus challenge than control plants. Several POLV- and JMJ14-regulated genes were found to be regulated in virus induced resistance in WT plants, with some of them poisoned to be expressed in early infection stages.

**Conclusions:**

A set of confident candidate genes directly regulated by the POLV and JMJ14 proteins during virus infection was identified, with indications that some of them may be regulated by multiple epigenetic modules. A subset of these genes may also play a role in the tolerance of WT plants to repeated, intermittent virus infections.

**Supplementary Information:**

The online version contains supplementary material available at 10.1186/s12870-024-04866-3.

## Introduction

Plants possess a robust innate immunity that comprises various anatomical adaptations as well as conserved general- and pathogen-specific receptors [1]. Pattern-triggered immunity (PTI) is a mechanism that uses specialized membrane receptors to recognize common molecular patterns from pathogens or host damage-derived compounds and is usually enough to control the majority of plant pathogens [2]. Some pathogens, on the other hand, deliver proteins that suppress or reduce PTI responses, resulting in disease. Conversely, plants may evolve specialized proteins to fight back, resulting in a complex and intricate evolutionary arms race [3]. These specialized intracellular nucleotide-binding oligomerization domain (NOD)-like resistance (NLR) proteins trigger a signaling cascade known as effector-triggered immunity (ETI), resulting in a strong defense mechanism. At infection sites, ETI defensive responses frequently involve cell death-like hypersensitivity response (HR), which is dependent on calcium-signaling cascades [4]. When stimulated by a pathogen, some plant defense genes are altered and switched to a pre-activated state, even in non-infected systemic tissues [5]. When plants are re-exposed to pathogens, defenses may be triggered faster and/or stronger, resulting in more efficient control than the initial onset, a phenomenon known as induced resistance [6]. As a result, these stress memory genes have increased transcription capacity and are said to be *primed* for expression, raising basal levels of defenses. Examples of genes that can be regulated by these mechanisms include defense-related WRKY transcription factors and pathogenesis-related (PR) proteins [6]. Importantly, defense genes in plants must be tightly regulated, as their expression above certain levels can result in growth arrest [7–9]. Therefore, strategies for expression control at the chromatin, RNA, and/or protein levels must be coordinated in order to regulate growth-defense trade-offs.

It has been observed in the last decade that epigenetics and other RNA silencing-related pathways play an important role in defense priming regulation [10]. The RNA-directed DNA methylation (RdDM) is a well-known pathway that targets transposable elements (TEs). RdDM is accomplished through the coordinated action of two plant-specific versions of RNA polymerase II. Guided by TE-derived small RNAs (sRNAs), a variety of epigenetic factors such as DNA and histone methyltransferases are recruited [11]. RdDM typically acts on the edges of TEs located in genic-rich regions of the genome, thereby creating and reinforcing a heterochromatin environment within euchromatin [12].

Histone modifications are also required to act in coordination with DNA methylation marks in the regulation of immunity. Histone H3 Lysine 4 trimethylation (H3K4m3), for example, is commonly associated with gene expression activation and transcriptional memory of some plant defense genes [13–19]. As a result of the deposition of H3K4m3 marks, histone demethylases are recruited to remove modifications typically associated with repression, such as H3K9m2 [20]. In non-stressed situations, however, deposition of repressive H3K9m2 and H3K27m3 marks, as well as removal of H3K4m3 activation marks, plays a critical role in the global suppression of NLR defense genes, preventing any toxic effects [7, 21, 22].

The epigenetic regulation of immunity genes against pathogens has been the subject of scarce investigations, and even fewer in RNA viral infections. It has been demonstrated that plants lacking specific RdDM and histone modification functions have altered susceptibility to various viruses [23–26]. We previously demonstrated that epigenetic pathways are required for mounting proper antiviral defenses in *Arabidopsis thaliana* infected with turnip mosaic virus (TuMV; species *Turnip mosaic virus*, genus *Potyvirus*, family *Potyviridae*) [23, 27]. In an effort to identify genes directly targeted by these proteins during infection, we analyzed here the transcriptomes of two *A. thaliana* epigenetics mutants, *polv* and *jmj14*, that displayed tolerance to the virus (i.e., less severe symptoms). As a component of the RdDM complex, the RNA polymerase V (POLV) protein is associated with DNA methylation processes related to TE regulation [28]. JUMONJI14 (JMJ14) is a histone demethylase protein that represses the expression of its direct targets by removing H3K4m3 activation marks [29–32]. Although JMJ14 has been found to be involved in TE repression [33], its binding to transcription factors NAC050 and NAC052 and telomeric repeat binding factors TRB1/2/3 also directs the protein to genes that are not associated with repetitive sequences [34–36]. Using a combination of transcriptome and genome occupancy data, we identified several candidate genes that are epigenetically regulated by POLV and JMJ14 during TuMV infection and demonstrated that some of them are associated with virus-triggered induced resistance effects in wild-type (WT) plants.

## Results

### The POLV and JMJ14 proteins regulate stress-related genes during TuMV infection

We previously found that *A. thaliana* genotypes with mutations in various RdDM and histone modification genes had altered TuMV infectivity when compared to WT plants [23, 27]. Here, mutants associated with a RdDM gene, *NRPE1*, and a histone modification gene, *JMJ14*, were chosen for further analysis. *NRPE1* codes for the largest subunit of PolV, whereas *JMJ14* for an H3K4m3 demethylase. In an effort to identify the genes directly affected by the mutations that lead to the previously observed viral tolerance, the transcriptomes of TuMV-infected WT, *polv*, and *jmj14* mutants, as well as their respective mock-inoculated controls, were obtained. Non-inoculated central rosette leaves were collected at 4 days post-inoculation (dpi), when no symptoms were observed, and at 7 dpi, just after symptoms appeared.

Because both the POLV and JMJ14 proteins act as repressors of gene expression via DNA methylation and histone modification, respectively, their direct targets are expected to be expressed at a higher level in mutants than in WT plants. As a result, we concentrated the analysis on the induced set of differentially expressed genes (DEGs). The induced genes at 4 and/or 7 dpi were combined and classified into four groups: WT responsive (infected WT vs. mock WT), mutant responsive (infected mutant vs. mock mutant), mutant-enhanced mock (mock mutant vs. mock WT), and mutant-enhanced infected (infected mutant vs. infected WT) (Fig. S1a and Table S1). The two categories labeled as “enhanced” contain genes that are more likely to be expressed in mutants compared to WT plants. This was done to enrich genes directly targeted by the two epigenetics proteins.

The induced genes in WT plants were enriched in several gene ontology (GO) categories related to metabolism, abiotic and biotic stress responses, with salicylic acid (SA) response being the most significant (Fig. S2). The majority of the mutant-induced genes were also responsive in WT plants (Fig. S1a). In the *polv* mutant, a total of 6157 induced genes were either enhanced in mock or infected tissues, and 5545 in the *jmj14* mutant (Fig. S1a and Table S1). The majority of the enriched GO categories were unique to each mutant (Fig. S2). These findings suggest that the POLV and JMJ14 proteins may directly or indirectly regulate several stress response pathways during TuMV infection, with little overlap.

### Identification of candidate genes that are directly regulated by the POLV and JMJ14 proteins during infection

**Fig. 1:**
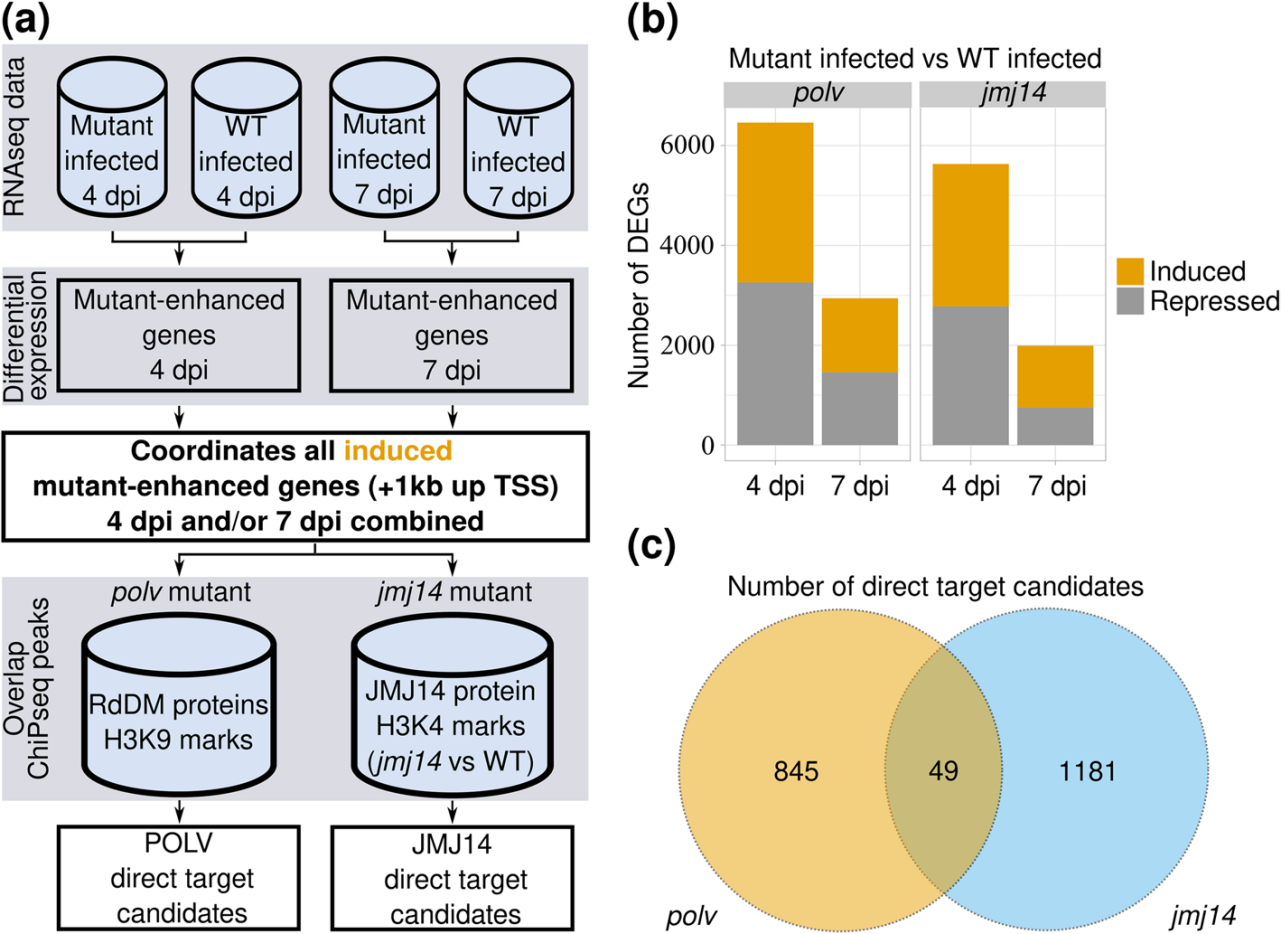
Identification of potential direct POLV and JMJ14 target genes. **a** Bioinformatics pipeline used for identifying targets. Transcriptome (RNAseq) data from mutant (*polv*, *jmj14*) and wild-type (WT) infected plants were processed separately at 4 and 7 days post-inoculation (dpi). Differential expression analysis was performed for each dpi using mutant infected samples as treatment and WT as control (mutant-enhanced infected group analysis). The genomic locations of all mutant-enhanced genes that were induced at 4 or 7 dpi, including 1 kilobase (kb) upstream of their transcriptional start sites (TSS), were extracted and overlapped with chromatin immunoprecipitation data (ChIP-seq). The genomic ranges of the POLV-regulated genes were compared to the combined chromatin occupancy of the RdDM-related proteins POLIV, POLV, DRD1, DMS3, RDM1 and H3K9m2 obtained from the NCBI GEO database. For the JMJ14-regulated set, genic locations were overlapped with JMJ14 ChIP-seq peaks and regions found to gain H3K4m3 marks in *jmj14* mutants when compared to WT plants. **b** The total number of differentially expressed genes (DEGs) discovered in the mutant-enhanced infected group prior to filtering with ChIP-seq data (adjusted *P* = 0.05). For each condition, three biological replicates were used, each with a pool of 12 plants. **c** Venn diagram depicting the total number of DEGs induced in the mutant-enhanced infected group with significant overlaps with ChIP-seq data

The augmented expression of genes in infected epigenetic mutants compared to WT infected plants is a common behavior observed in epigenetically regulated immunity genes [37]. As a result, we concentrated on the mutant-enhanced infected samples in an attempt to identify candidate genes that are directly targeted by the POLV and JMJ14 proteins (Fig. 1a). The number of DEGs in this category observed for both *polv* and *jmj14* mutants was higher at 4 than at 7 dpi (Fisher’s exact test: *P* < 0.0001). Gene expression reprogramming related to these epigenetic pathways was therefore more pronounced at early stages of infection on non-inoculated leaves than at fully established stages (Fig. 1b and Fig. S3). At 4 dpi, the number of induced and repressed DEGs distributed evenly across both mutant genotypes (Fisher’s exact test: *P* = 0.2289). However, at 7 dpi the number of induced DEGs was significantly enriched in *jmj14* compared to *polv* (Fisher’s exact test: *P* < 0.0001).

POLV and JMJ14 are epigenetic proteins that can repress genes directly by promoting DNA methylation and H3K4m3 removal, respectively, or indirectly by affecting numerous signaling cascades. Direct POLV targets are expected to have a higher chromatin occupancy of RdDM proteins and H3K9m2 marks compared to indirect targets. In turn, JMJ14 direct targets may have higher JMJ14 occupancy than indirect ones. It is expected that the targets of this histone demethylase will also be H3K4 hypermethylated in *jmj14* mutants compared to WT plants. Available chromatin immunoprecipitation followed by next generation sequencing (ChIP-seq) experiments for the RdDM-related proteins RNA Polymerase IV (POLIV), POLV, DEFECTIVE IN RNA-DIRECTED DNA METHYLATION 1 (DRD1), DEFECTIVE IN MERISTEM SILENCING 3 (DMS3), RNA-DIRECTED DNA METHYLATION 1 (RDM1) and H3K9m2 marks were obtained from the NCBI GEO database (Table S2). Processed peaks were overlapped with induced *polv*-enhanced genes in infected plants, including regions one kilobases (kb) upstream of the gene’s transcription start sites (TSS) (Fig. 1a). From a total of 4556 induced genes at 4 and/or 7 dpi in the infected *polv*-enhanced group (Fig. S1a), 894 had significant occupancy of RdDM proteins or H3K9m2 marks. These overlapping genes could be potential direct targets of the POLV protein during infection (Fig. 1c and Table S3).

Genome occupancy data for the JMJ14 protein and H3K4m2/m3 marks in *jmj14* mutants and WT plants were also obtained and analyzed from published studies (Table S2). Genomic ranges of all induced genes at 4 and/or 7 dpi enhanced in *jmj14*-infected samples (plus 1 kb upstream from the TSS) were overlapped with JMJ14 ChIP-seq peaks and regions found to gain H3K4m3 marks in *jmj14* mutants. From a total of 3,233 genes in the *jmj14*-enhanced infected group (Fig. S1a), 1230 were found to overlap with JMJ14 or H3K4 hypermethylation peaks (Fig. 1c and Table S3). The overlap between direct candidates was minimal, with only 49 genes shared by both groups (Fig. 1c and Table S3).

**Fig. 2:**
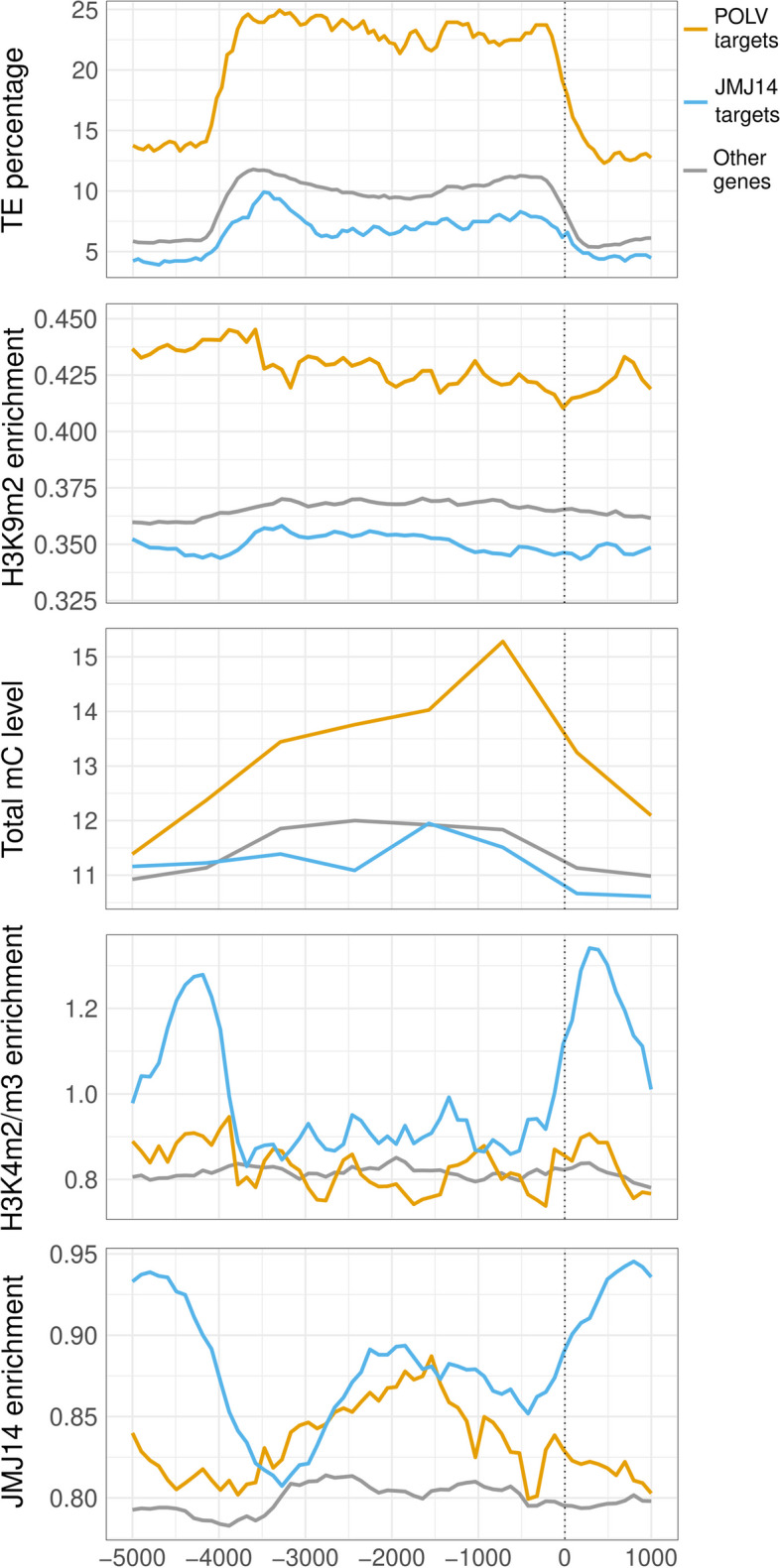
Metaplots displaying the percentage of transposon elements (TE), mean enrichment of H3K9m2, H4K4m2/m3, and JMJ14 proteins, and total methylcytosine (mC) levels around transcriptional start sites (TSS) of candidate genes to be directly regulated by POLV (*n* = 894) or JMJ14 (*n* = 1,230). The values were calculated in 60 base pair windows. Ranges upstream and downstream of the TSS are represented by negative and positive numbers in the Xabscissa- axis, respectively. The TSS is indicated by dashed lines

To validate the dataset of genes regulated by the two epigenetic proteins, profiles of different features were computed around the TSS (5 kb upstream and 1 kb downstream) of each one using ChIP-seq and whole genome bisulfite sequencing (WGBS-seq) data obtained from public databases (Table S2 and Fig. 2). As expected, direct POLV targets had a higher percentage of TEs and TE-related H3K9m2 and methylcytosine (mC) marks around their TSS than other genes (Fig. 2). In contrast, the set of JMJ14-regulated genes had lower levels of these same features around their TSS than the control set. This indicates that genes found to be augmented in infected *polv* mutants but not the *jmj14* ones are probably controlled by TE-related mechanisms. The set of JMJ14-regulated genes had two main peaks of hypermethylated H3K4m2/m3 marks in *jmj14* mutants close to 4 kb upstream and 500 bp downstream of the TSS (Fig. 2). These peaks coincided with JMJ14 protein enrichment regions in WT plants (Fig. 2). This means that in mutant *jmj14* plants, the regions of JMJ14 chromatin binding coincide with an average increase in H3K4 hypermethylation for this specific dataset. The set of POLV-regulated or non-regulated genes, on the other hand, showed no gain of H3K4 activation marks in *jmj14* mutants in these same positions (Fig. 2). JMJ14 binding was increased around 2 kb upstream of the TSS in both POLV- and JMJ14-regulated genes, but this was not associated with increases in H3K4 marks in *jmj14* mutants for the POLV dataset (Fig. 2). Other H3K4 demethylases could be redundantly removing these marks in these regions in the mutant. The profiles showed therefore that each dataset contained the expected marks for their respective pathways, indicating that they are likely enriched in true direct targets of these epigenetics proteins. These results collectively indicate that approximately 20% and 40% of all induced genes at 4 and/or 7 dpi, respectively, are candidates for direct regulation by POLV and JMJ14 during infection.

### The majority of the POLV- and JMJ14-regulated candidate genes are protein-coding and dynamically regulated during infection

**Fig. 3:**
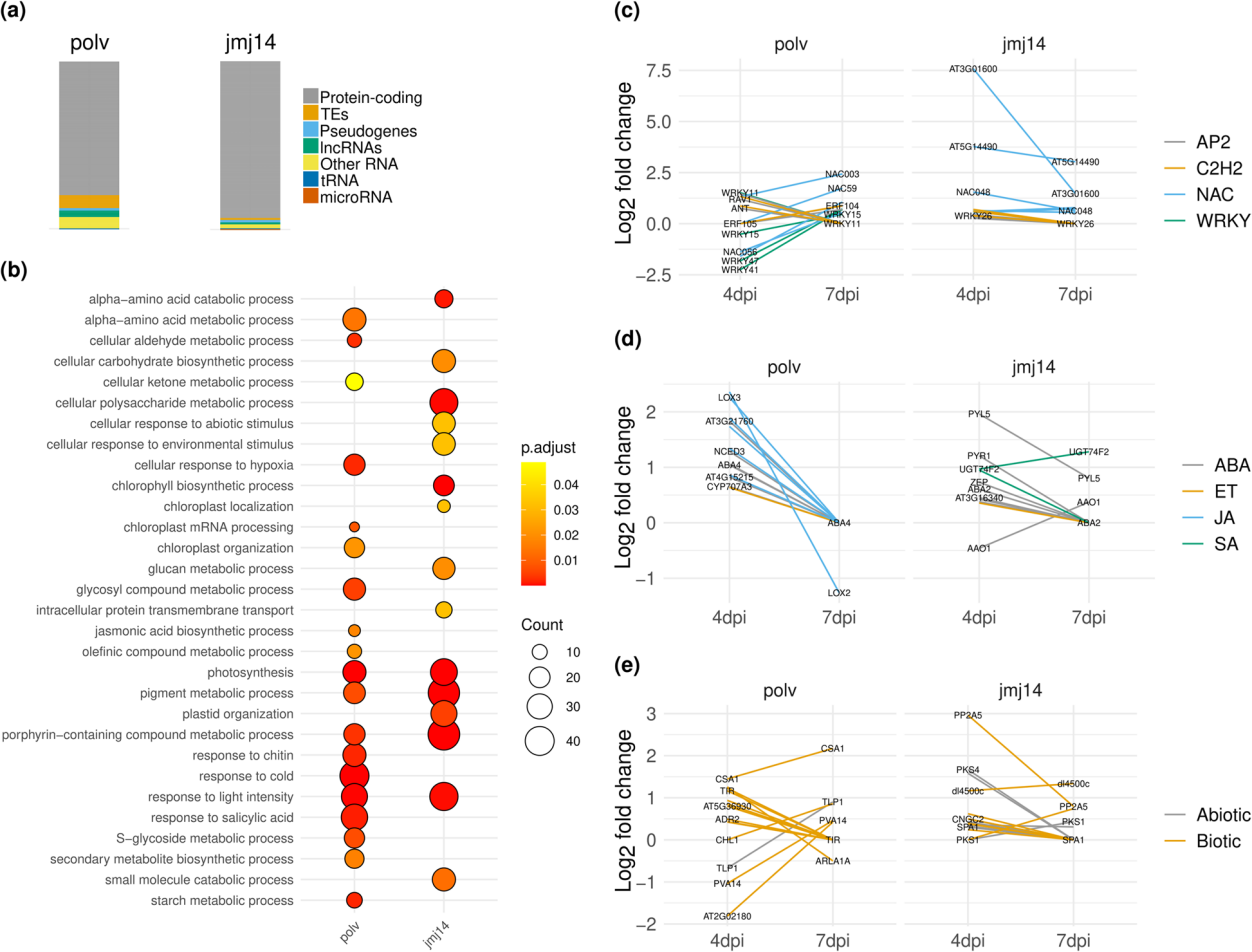
Biological characteristics of genes that are predicted to be directly targeted by the POLV and JMJ14 proteins during TuMV infection. Direct targets consisted of genes that were induced in *polv* or *jmj14*-infected mutants relative to infected wild-type plants at 4 and/or 7 days post-inoculation (dpi) and that overlapped with particular epigenetic marks or proteins. **a** Gene types based on *A. thaliana* RTD2 annotation. Transposable elements (TEs); long non-coding RNAs (lncRNAs); and transfer RNAs (tRNAs). **b** Gene ontology analysis of direct POLV and JMJ14 targets. The size of a circle represents the degree of enrichment, whereas color heat maps represent adjusted *P* values (p.adjust). (c-e) Transcriptional profiles of selected transcription factor genes (**c**), phytohormones (**d**), and stress-related genes (**e**)

The vast majority of the candidate genes found to be directly regulated by POLV and JMJ14 were protein-coding genes, but non-coding RNAs and TEs were also observed (Fig. 3a). Interestingly the distribution of candidate genes among the categories listed in Fig. 3a were significantly different between POLV and JMJ14 (χ^2^ = 49.485, 7 d.f., *P* < 0.0001), with an enrichment of protein-coding RNAs in the case of JMJ14 and of TEs in the case of POLV. The major GO categories enriched in POLV-regulated genes were related to biotic and abiotic stress responses. Although some abiotic-related GOs were also enriched in JMJ14-regulated genes, the majority were involved in metabolism regulation (Fig. 3b). Both datasets of direct candidates contain Mapman annotation bins related to transcriptional factors (TF), phytohormones, and immune regulation (Fig. 3c-e). Several members of the TF families NAC and WRKY were downregulated at 4 dpi in *polv*-infected samples, but upregulated at 7 dpi when compared to the level of expression in infected WT plants (Fig. 3c). However, higher expression in mutant infected plants than WT infected at 4 dpi and similar levels in both conditions at 7 dpi, was generally observed for various genes associated with the phytohormone classes abscisic acid (ABA), ethylene, jasmonic acid (JA), SA and stress-related genes. This indicates that epigenetic regulation is especially important for early expression of defense genes in non-inoculated leaves (Fig. 3d, e).

**Fig. 4:**
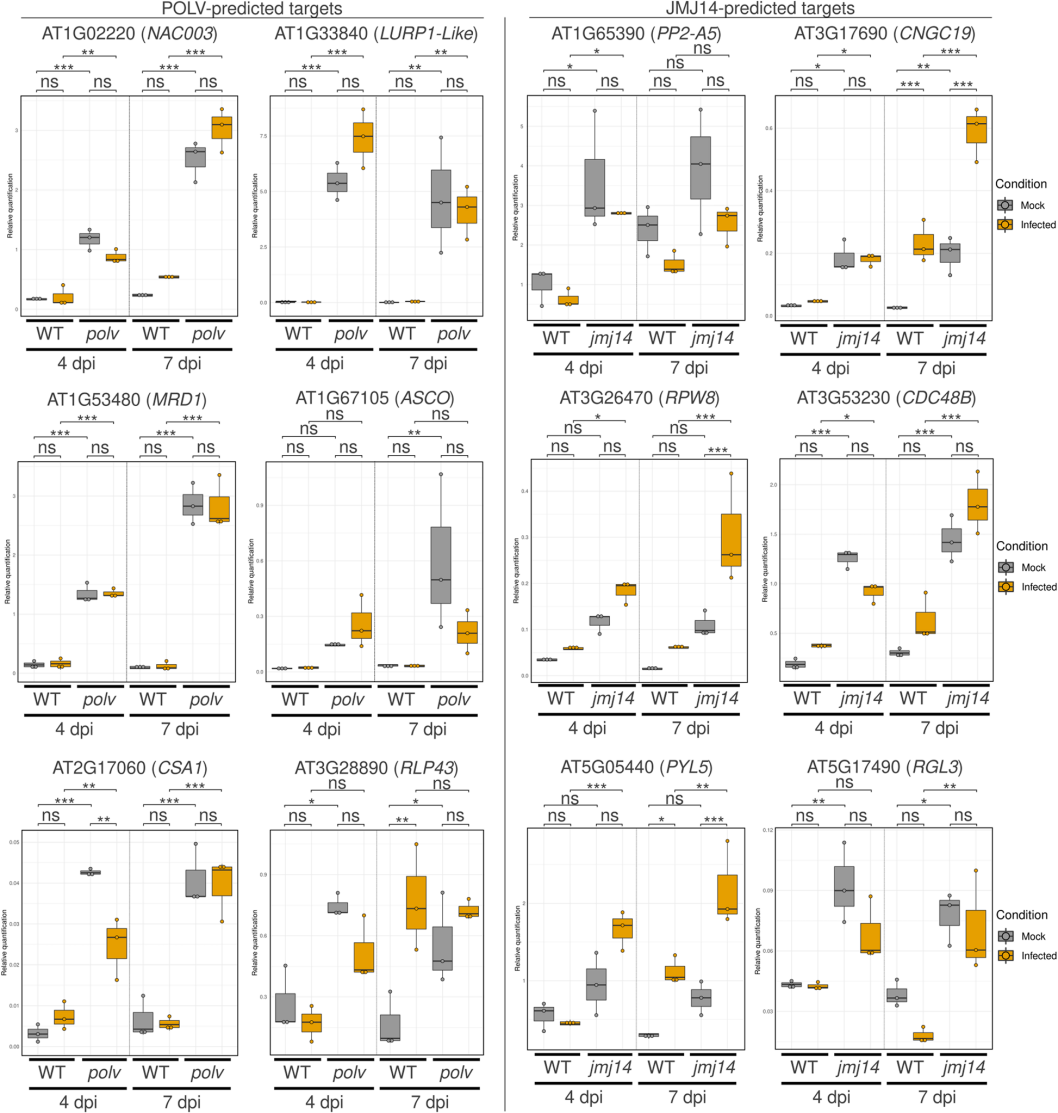
RT-qPCR analysis of selected genes predicted to be directly targeted by the POLV and JMJ14 proteins during TuMV infection. Direct targets were genes that were induced in mutant *polv* or *jmj14*-infected mutants compared to wild-type infected plants at 4 and/or 7 days post-inoculation (dpi) and overlapped with specific epigenetic marks or proteins. POLV predicted targets (*NAC003*, *LURP1-LIKE*, *MRD1*, *ASCO*, *CSA1*, *RLP43*, and *PP2-A5*) and JMJ14 predicted targets (*CNGC19*, *RPW8*, *CDC48B*, *PYL5,* and *RGL3*) were quantified at 4 and 7 dpi (TuMV or mock inoculation). For the relative quantification, *A. thaliana* endogenous genes *SAND* and *PP2A* were used as references. Pairwise *t*-test comparisons were performed with Bonferroni correction tests; ***P < 0.001; **P < 0.01; *P < 0.05; ns., not significant

Six POLV-regulated genes and six JMJ14-regulated genes were chosen for reverse transcription quantitative polymerase chain reaction (RT-qPCR) validation based on function and fold change differences in mutant infected vs. WT infected (Fig. 4). All tested genes, except *ALTERNATIVE SPLICING COMPETITOR* (*ASCO*) and *RECEPTOR LIKE PROTEIN 43* (*RLP43*), confirmed enhanced expression in infected mutants at 4 and/or 7 dpi when compared to infected WT plants (Fig. 4). With the exception of the ABA-receptor gene *PYRABACTIN RESISTANCE 1-LIKE 5* (*PYL5*) and the gene coding for the *RESISTANCE TO POWDERY MILDEW 8* (*RPW8*)-domain containing protein (*RPW8* hereafter), all of the other tested genes showed augmented expression in mock mutant plants at either 4 or 7 dpi (Fig. 4). This suggests leaky expression in the mutants prior to infection when compared to WT uninfected plants. When comparing mutant infected plants to mock mutant plants, genes coding for RPW8 and the calcium channel-related *CYCLIC NUCLEOTIDE GATED CHANNEL 19* (*CNGC19*), both predicted as JMJ14 targets, an increased expression in mutant infected plants was observed at both time-points (Fig. 4). Although non-infected plants have similar levels of *PYL5* expression, *jmj14* infected plants have a much stronger induction of this gene than WT infected plants (Fig. 4). These data collectively indicate that several genes involved in various aspects of immune responses are dynamically regulated during infection.

### Virus-responsive genes may be controlled by distinct epigenetic modules

To increase confidence that the selected panel of genes are indeed regulated by epigenetic mechanisms, their sequences were analyzed in the Plant Chromatin State Database (PCSD). In PCSD, a series of 36 epigenetic states that are commonly found correlated in the *A. thaliana* genome were identified based on the integration of publicly available epigenomic data [38] (Fig. S4). Three of the six POLV-regulated selected genes had TE-related epigenetic states associated with their promoter sequences, including the *NAC DOMAIN CONTAINING PROTEIN 3 (NAC003*) transcription factor, the *ASCO* long non-coding RNA (lncRNA), and the NLR gene *CONSTITUTIVE SHADE-AVOIDANCE 1* (*CSA1*) (Fig. S4). The *CSA1* gene’s promoter also contained bivalent marks, including repression H3K27m3, activation H3K4m2, and the histone variant H2A.Z (Fig. S4). The promoter of the defense-related POLV-regulated gene *LATE UPREGULATED IN RESPONSE TO HYALOPERONOSPORA PARASITICA-ONE-LIKE* (*LURP1-LIKE*) was also found to have bivalent and H3K27m3 repression states (Fig. S4). None of the JMJ14 selected genes were linked to epigenetic states related to TE regulation, providing more evidence that they are not regulated by RdDM-related mechanisms (Fig. S4). With the exception of *PYL5* and gibberellin-associated *RGA-LIKE PROTEIN 3* (*RGL3*) genes, where only non-H3K4m-related accessible DNA states were found, the promoter sequences of the remaining four JMJ14 selected genes, the defense-related *PHLOEM PROTEIN 2 A5* (*PP2-A5*), *CNGC19*, *RPW8*, and the chaperone *CELL DIVISION CYCLE 48B* (*CDC48B*) displayed both bivalent and activation marks (Fig. S4).

**Fig. 5:**
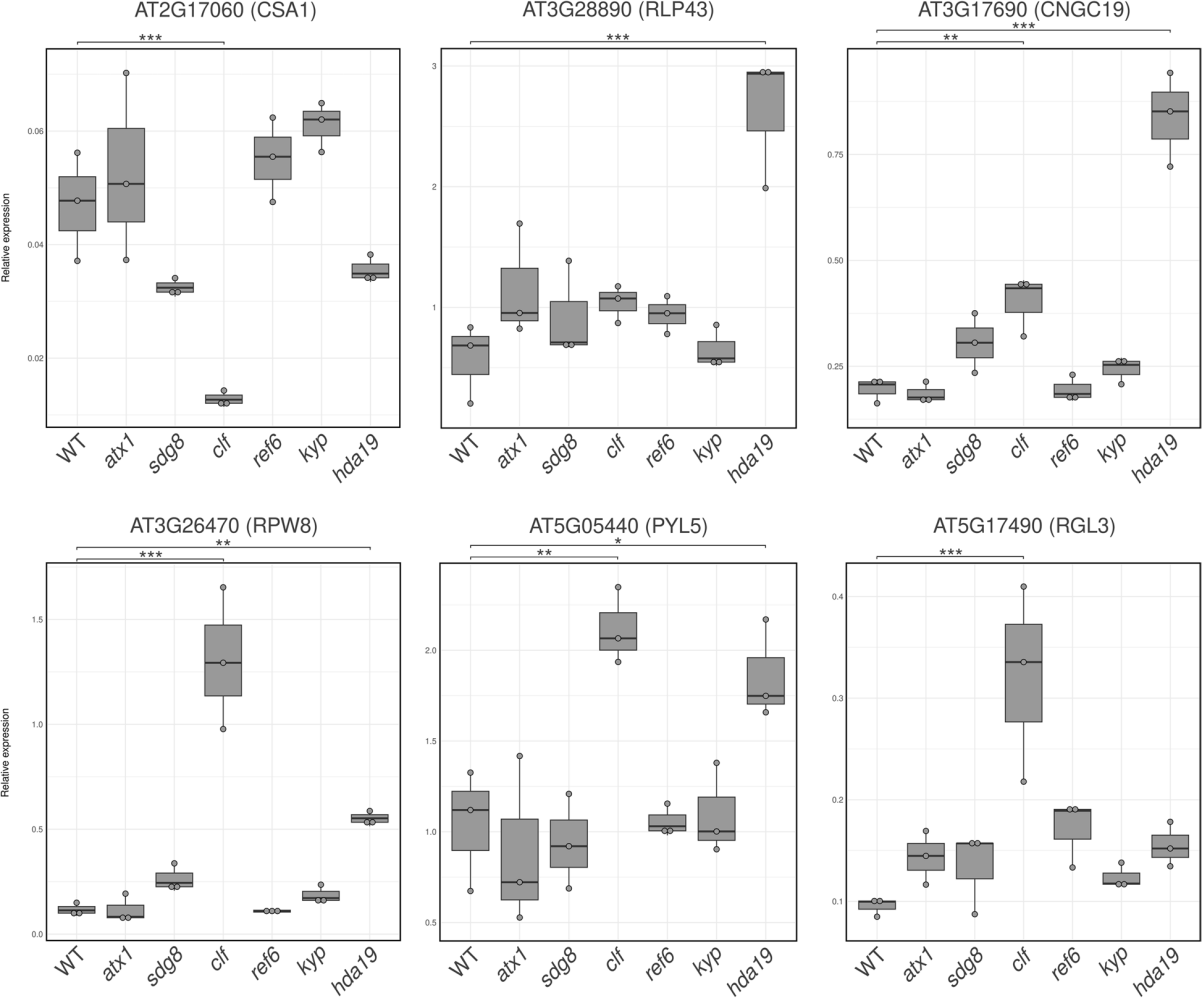
Expression of selected genes across different epigenetic mutants. The expression of the POLV predicted targets (*CSA1* and *RLP43*) and JMJ14 predicted targets (*CNGC19*, *RPW8*, *PYL5*, and *RGL3*) was quantified by RT-qPCR in the epigenetic mutants *atx1* (deficient in H3K4 methylation), *sdg8* (deficient in H3K36 methylation), *clf* (deficient in H3K27 methylation), *ref6 *(deficient in H3K27 demethylation), *kyp* (deficient in H3K9 methylation) and *hda19* (deficient in histone deacetylation). *A. thaliana* endogenous genes *SAND* and *PP2A* were used as references for relative quantification. Pairwise *t*-test comparisons were performed with Bonferroni correction tests; ***P < 0.001; **P < 0.01; *P < 0.05; ns., not significant

The presence of different kinds of marks in the promoter regions of the selected genes suggested that additional epigenetic modules may possibly be involved in their regulation. To directly test this hypothesis, the expression of the POLV-controlled genes *CSA1* and *RLP43*, as well as the JMJ14-controlled genes *CNGC19*, *RPW8*, *PYL5*, and *RGL3*, was examined in six additional epigenetic mutants. Plants defective in the methyltransferase genes *ARABIDOPSIS TRITHORAX 1* (*ATX1*), *CURLY LEAF* (*CLF*), *KRYPTONITE* (*KYP*), and *SET DOMAIN GROUP 8* (*SDG8*), associated with H3K4, H3K36, H3K27 and K3K9 methylation marks, respectively, were used in this assay. The H3K27 demethylase gene *RELATIVE OF EARLY FLOWERING 6* (*REF6*) and the histone deacetylase gene *HISTONE DEACETYLASE 19* (*HDA19*) were also included. All of the selected pathways have previously been linked to the regulation of defense genes [39]. Among the examined mutant genotypes, expression analysis indicated that the CLF and HDA19 proteins may play the most significant role in the regulation of the selected genes. Genes *CNGC19*, *PYL5* and *RLP43*, *RPW8* were induced in *hda19* mutants relative to WT plants (Fig. 5). This is consistent with HDA19 protein’s predicted restrictive action in its targets due to the removal of activation acetylation marks. In the *clf* mutant background, *CNGC19*, *CSA1*, *PYL5* and *RPW8*, were also dysregulated, suggesting that H3K27m marks may also be necessary for their regulation (Fig. 5). Contrary to expectations, *CSA1* expression was lower in *clf* mutants than in WT plants, suggesting that this gene may be repressed via alternative mechanisms in the absence of CLF (Fig. 5). The expression of all six examined genes was not significantly affected in the lines *kyp*, *ref6* and *sdg8*, when compared to WT plants (Fig. 5). This could imply that the selected virus responsive genes are not targeted by these epigenetic proteins, or that their functions are performed by other family members in their absence.

Overall, the selected genes’ overlap with epigenetic proteins or marks (Fig. 1 and Table S3), their altered expression in epigenetic mutants (Figs. 4 and 5), and the presence of bivalent, activation, and repression epigenetic marks in their promoters (Fig. S4) all increase the likelihood that they —and the majority of the other identified candidates- are indeed regulated by epigenetic pathways during virus infection.

### A subset of candidate genes regulated by POLV and JMJ14 are also regulated in WT plants submitted to repeated virus infection

**Fig. 6:**
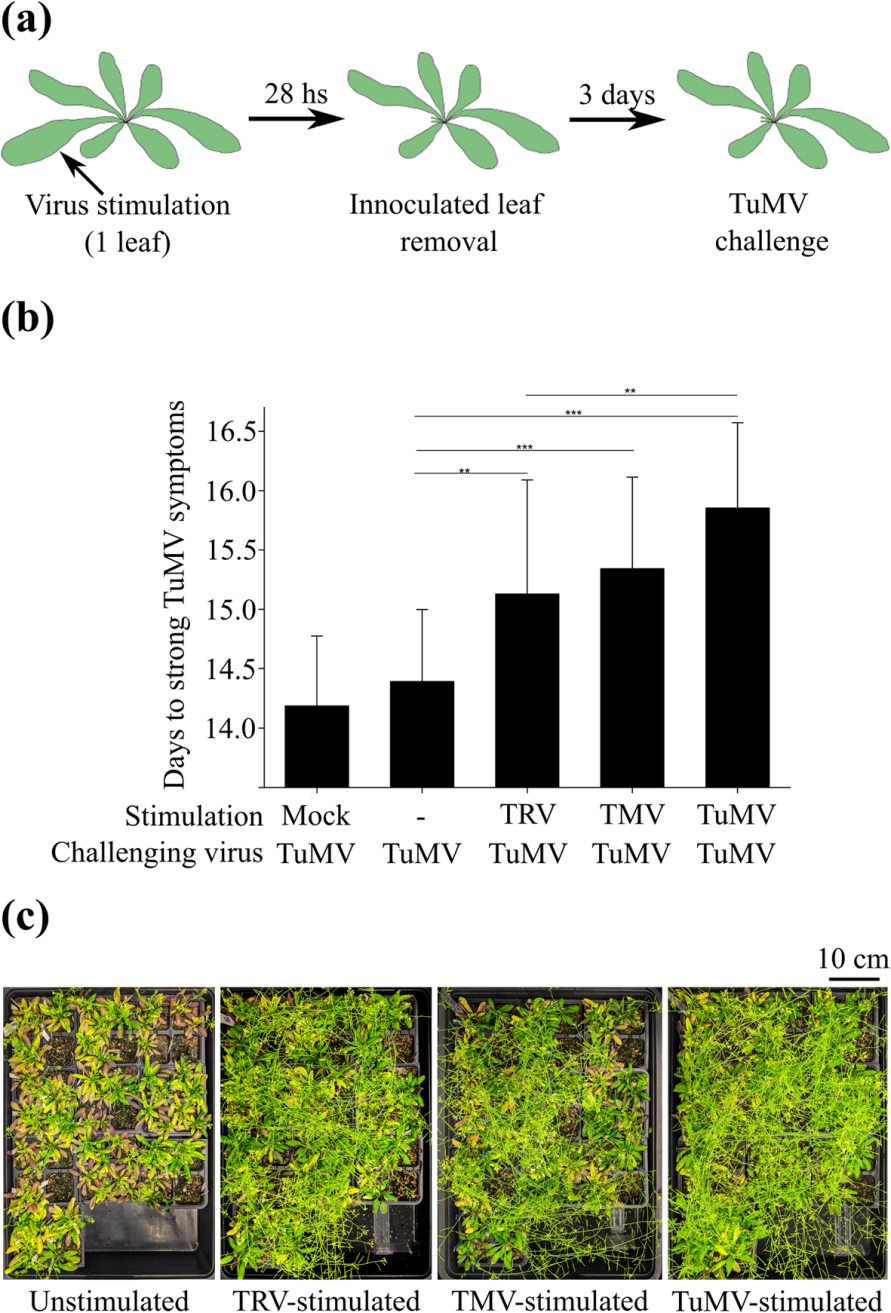
Wild-type *A. thaliana* plant susceptibility to repeated virus stresses. **a** Experimental design. Batches of 88 three-week-old plants were inoculated with the stimulating viruses TRV, TMV, ROS-tagged TuMV, or mock-inoculated, and 26 - 28 h later, the inoculated leaves were removed to prevent virus spread. Three days after leaf removal, stimulated plants were either challenged with TuMV (*n* = 78) or mock-inoculated (*n* = 10). A control batch of plants was also challenged with TuMV after being left without any manipulation (mocking or leaf removal) during the stimulation step. **b** The number of days it took each plant to develop strong symptoms (all rosette leaves with clear yellowing symptoms) following TuMV inoculation. The stimulation condition included the following treatments performed prior to leave removal: mock inoculation, no manipulation (-), or inoculation with TRV, TMV, or ROS-tagged TuMV. TuMV was inoculated three days after leaf removal in all cases. The results are based on six experimental replicates, each with 88 plants for each condition. Pairwise *t*-tests: ***P < 0.01; **P < 0.05. **c** Experimental replicate illustrating a significant visual difference in symptomatology between unstimulated and TRV-, TMV-, or TuMV-stimulated plants 21 days after TuMV challenge

It is known that some stress genes in plants are epigenetically regulated in response to recurrent stress situations. We wondered if some of the POLV- and JMJ14-regulated genes could have a biological role in WT plants under sequential viral stress. Plants were inoculated with a stimulating virus, and leaves were removed before virus spread to non-inoculated tissues (Fig. 6a and Fig. S5). After three days of recovery, a second challenging virus was inoculated, and symptoms were recorded for 21 days (Fig. 6b). Plants that had not been inoculated or had been mock-inoculated served as controls. TuMV was used as the second challenging virus in all cases. To determine if phylogenetic distance to TuMV influenced the results, tobacco mosaic virus (TMV; *Tobamovirus*, *Virgaviridae*), tobacco rattle virus (TRV; *Tobravirus*, *Virgaviridae*), and TuMV itself were used as stimulating viruses in separate experiments.

The level of TuMV symptom severity in plants was the same whether they were mock inoculated or left untouched in the stimulation step (Fig. 6b). Plants stimulated with any of the three viruses were more tolerant to subsequent TuMV infection than unstimulated plants (Fig. 6b), despite the high variability among the six full factor experimental replicates performed. Unstimulated plants developed stronger symptoms faster, as defined by the presence of clear yellowing in all rosette leaves (Fig. 6c). Interestingly, stimulation with TuMV made them more tolerant to later TuMV challenge than stimulation with TRV, indicating that the phylogenetic proximity between the stimulating and the challenging stresses was relevant for the outcome of the induced resistance phenotype. In some cases, the differences in TuMV tolerance between stimulated and unstimulated plants became visually prominent in late infection time-points (Fig. 6c).

**Fig. 7:**
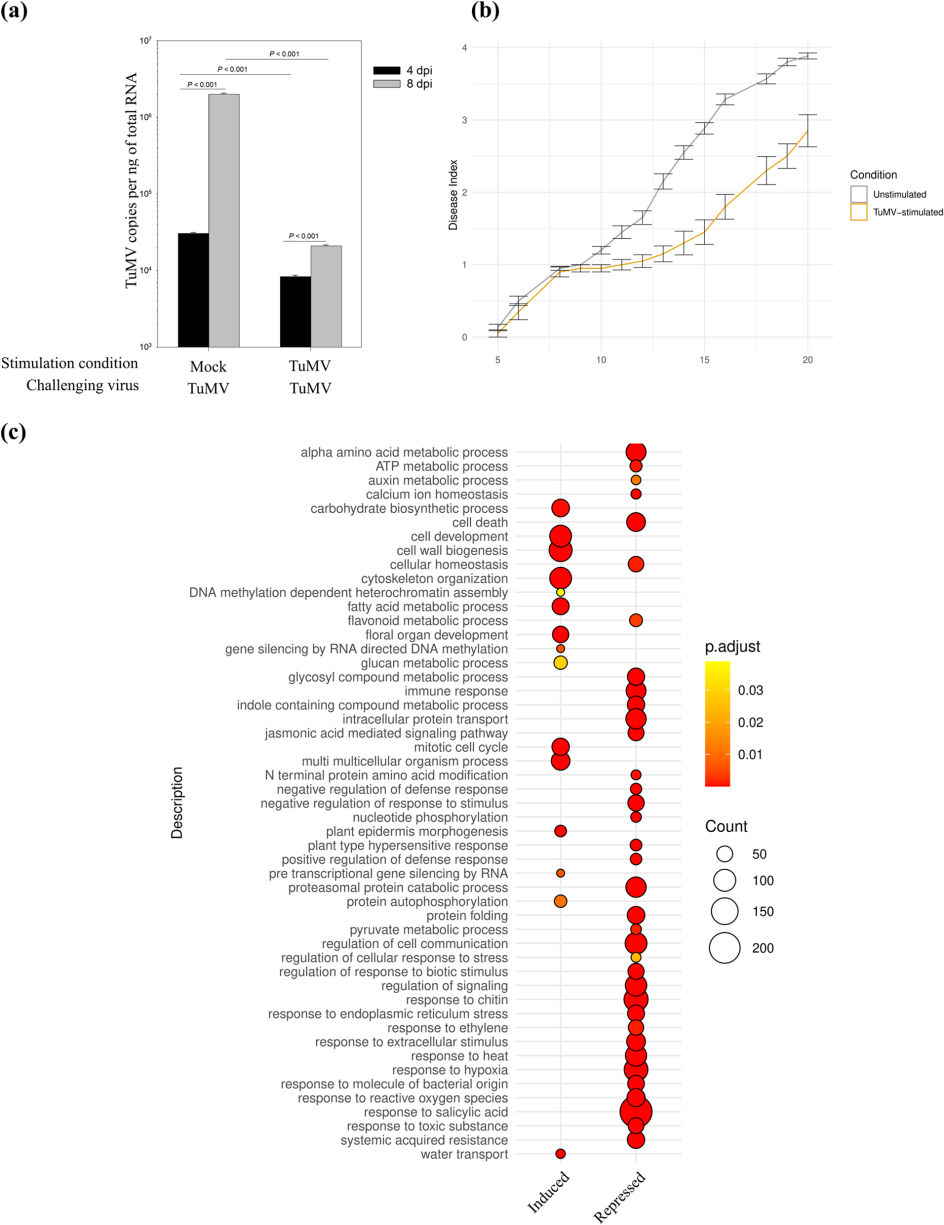
Virus quantification and transcriptome analysis of wild-type *A. thaliana* plants subjected to multiple virus stresses. **a** Absolute RT-qPCR analysis of TuMV load in samples that had been mock- or ROS-TuMV-stimulated prior to TuMV challenge. Inoculated leaves were removed 26-28 hours later to prevent virus spread or account for mechanical stress responses. In both conditions, TuMV was inoculated three days after leaf removal. For each condition, six individual plants were sampled four and eight days after the second TuMV inoculation (dpi). **b** TuMV symptom severity progression in unstimulated (*n* = 60) and TuMV-stimulated (*n* = 20) plants. Symptoms were scored daily using the scale described in the Methods section, with higher numbers indicating more severity. **c** Gene ontology analysis of induced and repressed genes. DESEq2 was used for differential expression analysis, with TuMV-stimulated plants as treatments and mock-stimulated plants as controls. The size of the circles represents the degree of enrichment, whereas the colors of the heat maps represent the adjusted *P* values (p.adjust)

To compare the dynamics of gene expression patterns and maximize the chances of finding virus memory genes, an experimental replicate with high TuMV tolerance due to TuMV stimulation was chosen for further investigation. Plants that had been stimulated with TuMV prior to challenge showed a significantly lower virus load four days after the challenge (Fig. 7a). This difference in virus load between conditions was even greater at 8 dpi, indicating that TuMV infection progressed more slowly in stimulated plants. Indeed, TuMV viral titers in stimulated plants at 8 dpi were comparable to those in unstimulated samples at 4 dpi, indicating that the two conditions were in different stages of infection at 8 dpi. The observed viral load differences during the early and middle stages of infection corresponded well with symptomatologic data (Fig. 7b). Disease severity increased more slowly in TuMV-stimulated plants than in unstimulated ones after about 10 dpi (paired-sample Wilcoxon test: *P* = 0.0001221). This suggests that the stimulation affected the expression of immunity-related genes, resulting in less viral accumulation and an arrest in symptom progression. Four unstimulated and TuMV-stimulated individual plants were chosen for transcriptome analysis at 8 dpi. As controls, three biological replicates of plants that were mock-inoculated in both the stimulation and challenging steps (mock plants) were included. This time point was chosen due to the greater differences in virus load between stimulated and unstimulated samples, as both of which had very low virus titers at 4 dpi. In the principal component analysis (PCA), both unstimulated and stimulated samples showed clear separation when compared to mock plants with no virus (Fig. S6a-b). We concentrated on a direct comparison between unstimulated and stimulated plants to find memory genes. When both conditions were compared, a general clear separation in PCA was observed, indicating that stimulation conditions influenced later infection expression patterns. However, one outlier was observed among the four biological replicates of unstimulated samples and another among the stimulated samples (Fig. S6c). The outliers were therefore removed, and the differential expression analysis was carried out with the remaining three biological replicates (Table S4).

The major unique GO categories in the set of induced genes were related to development and metabolism when the low virus titer TuMV-stimulated plants were used as treatments and the high virus titer unstimulated plants were used as controls (Fig. 7c). Induced genes were also found to be overrepresented in three categories related to heterochromatin assembly and RdDM-related processes (Fig. 7c). The majority of stress-related GO categories were only found in the set of repressed DEGs (Fig. 7c), reflecting the significantly lower virus load in stimulated samples at 8 dpi (Fig. 7a and Table S4).

**Fig. 8:**
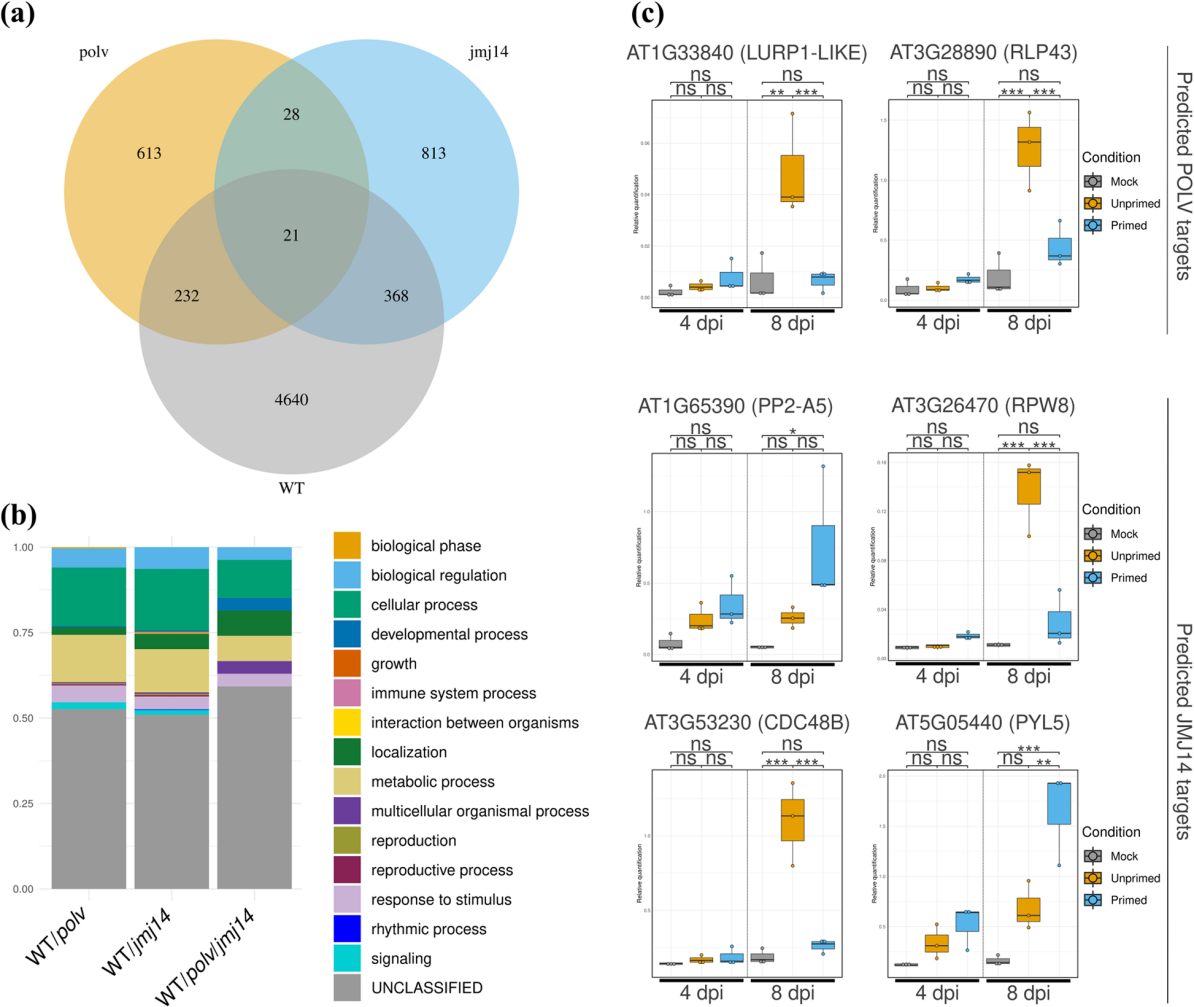
Expression and biological analysis of POLV and JMJ14-regulated genes that are also stimulated in wild-type (WT) plants. **a** Venn diagram including a list of *A. thaliana* genes that are possibly directly controlled by POLV, JMJ14, and repeated stress in WT plants. The POLV and JMJ14 sets included genes that were activated in *polv* or *jmj14*-infected mutants compared to WT infected plants at 4 and/or 7 days post-inoculation (dpi) and overlapped with specific epigenetic marks or proteins. The WT set consisted of genes that were regulated in TuMV-infected plants, when comparing plants that were previously stimulated with a ROS-tagged TuMV to plants that were only mock-inoculated. **b** Biological process types at intersections of genes regulated in WT and mutant plants. The Panther database was used for gene list analysis. **C** RT-qPCR study of selected genes in WT plants subjected to repeated viral stresses and control plants. Plants were either mock-inoculated or infected with a ROS-tagged TuMV, and the inoculated leaves were removed 26-28 hours later. Three days after leaf removal, plants were inoculated with TuMV. Mock samples were mock-inoculated before and after leaf removal. Unstimulated samples were mock-inoculated before leaf removal and subsequently inoculated with TuMV. Stimulated samples were TuMV-inoculated before leaf removal and then inoculated again with TuMV. Each dot represents a biological replicate, which consists of individual plants collected four or eight days following the TuMV challenge phase. Two of the six selected genes (*LURP1-LIKE* and *RLP43*) were predicted to be regulated by POLV during virus infection, whereas four (*PP2-A5*, *RPW8*, *CDC48B*, and *PYL5*) were predicted to be controlled by JMJ14. *A. thaliana* endogenous genes *SAND* and *PP2A* served as controls for the relative quantification. Bonferroni correction tests were used for *t*-test pairwise comparisons; ***P < 0.001; **P < 0.01; *P < 0.05; ns., not significant

Among the 5261 DEGs between stimulated and unstimulated plants, 232 also appeared on the list of genes predicted to be directly controlled by POLV, 368 on the list of genes predicted to be JMJ14-regulated, and 21 on both lists (Fig. 8a and Table S5). Although there were no GO enrichment categories among these shared datasets, genes related to response to stimulus were present in all of them (Fig. 8b). Genes involved in organism interaction and immune responses were also found in the WT and JMJ14 groups. Six genes that had previously undergone RT-qPCR testing in mutant samples (Fig. 4) and that also appeared on the list of regulated genes in stimulated wild type plants (Table S5) were chosen for additional expression quantification analysis (Fig. 8c). At 4 dpi, a non-significant tendency to induction in stimulated plants was observed for all tested genes, including the defense-related *LURP1-LIKE* and *PP2-A5*, the PTI receptor *RLP43*, the defense-related *RPW8*, the chaperone *CDC48B*, and the ABA-receptor *PYL5* (Fig. 8c). Only samples collected at 8 dpi, which were the same ones used for the transcriptome analysis, showed significant differences, though. In four genes (*CDC48B*, *LURP1-LIKE*, *RLP43*, and *RPW8*), unstimulated samples showed a significant induction when compared to mock or stimulated samples, but there were no differences between stimulated and mock samples (Fig. 8c). This is consistent with the general repression of gene expression observed in stimulated samples when compared to unstimulated samples (Figs. 7c and 8c). Induction of the *PYL5* gene was observed in stimulated samples versus unstimulated samples, despite the fact that the former has significantly lower virus titer than the latter (Figs. 7a and 8c). This suggests that some of the identified POLV- and JMJ14-regulated genes during infection are dynamically regulated in WT plants exposed to repeated virus stress, with some of them being poisoned to be expressed at earlier stages of the infection.

## Discussion

Plants require defenses to avoid or combat pathogens, but they must also maintain tight control over their immune systems to avoid compromises in growth or interaction with beneficial organisms. Epigenetics is known to fine-tune defense gene expression but knowledge in virus contexts is limited. We found that approximately 20% and 40% of all induced genes in the *polv* and *jmj14* mutants at 4 and/or 7 dpi are candidates for direct regulation by these epigenetic proteins during infection, respectively (Table S1 and S3). This is consistent with previous studies indicating that approximately 15% of genes deregulated in the DNA demethylase *ros1* mutant are likely to be directly regulated by this protein [40, 41].

One limitation of the approach used to identify putative direct targets is that all ChIP-seq data from epigenetic proteins and marks were obtained in non-infected samples and, in some cases, from flower tissues available in databases (Table S2). Targets that would be regulated specifically or more strongly during virus infection processes may thus be missed, as was the case with previously identified JMJ14-modulated genes in bacterial infection contexts [42]. Despite this limitation, each dataset of POLV and JMJ14 putative targets contained the expected marks for their respective pathways, including enrichment of TEs, H3K9m2 and total mC marks around the TSS of the POLV-regulated ones and JMJ14 protein and propensity to H3K4m3 deposition in *jmj14* mutants in the JMJ14-regulated ones (Fig. 2), indicating that they are likely enriched in true direct targets of these epigenetics proteins. Although the vast majority of the candidate genes found to be directly regulated by POLV and JMJ14 during infection were protein-coding genes (Fig. 3a), consistent with their roles in TE-related DNA methylation and genic histone modifications, the overlap between the two datasets was very low (Fig. 1c). The majority of identified genes were more expressed in infected mutant plants than in infected WT plants at 4 dpi than at 7 dpi (Fig. 3c-e), indicating however that earlier induction due to virus stress in non-inoculated leaves may be a key factor in the tolerance observed in both *polv* and *jmj14* mutant plants.

Several genes associated with stress were identified as potential direct targets of POLV and/or JMJ14 (Fig. 3b-e and Table S3). Genes related to defense, phytohormones, and transcription factors were among the 12 chosen for a more in-depth analysis. All selected genes were more likely to be expressed in infected and/or non-infected conditions in epigenetically-deficient plants than in WT plants (Fig. 4). This confirms that under normal conditions, their expression is suppressed by mechanisms that may require epigenetic modifications in WT plants. Relevant marks were also found in the promoter sequences of selected genes based on a database integrating genome-wide maps of chromatin states, reinforcing their possible epigenetic regulation during infection (Fig. S4). Bivalent H2A.Z/H3K4m2/H3K27m3 marks, which are frequently associated with stress-memory genes [43], were found in the promoters of the POLV-regulated NLR gene *CSA-1* and the defense related gene *LURP1-LIKE*. The promoter of the JMJ14-regulated defense-related gene *RPW8* also showed induction and repression states.

Three of the selected genes (*CNGC19*, *PYL5* and *RPW8*), all of which are JMJ14-regulated candidates, were shown to be unregulated in both *clf* and *hda19* mutants (Fig. 5). The JMJ14-regulated gene *RGL3* was likewise induced in *clf* mutants, but with a non-significant induction in the *hda19* genotype. CLF is a member of the widely conserved multicomponent Polycomb repressive complex 2 (PRC2), which is necessary for the epigenetic control of genes involved in numerous biological processes [44]. The PRC2 requires prior histone deacetylation of its targets in order to perform its repressive effects, and HDA proteins, including HDA19, have been reported in association with the complex [45]. Indeed, it has been demonstrated that HDA19 and its homologue HDA9 are partly needed for the recruitment of CLF to the cold-regulated flowering-related gene *FLOWERING LOCUS C* [46]. Similarly, levels of H3K4 and H3K27 marks in PRC2 targets must be precisely balanced, and the H3K4 JMJ14 has been linked to PRC2 components [47]. The observed change in expression patterns of these virus-responsive genes in *clf*, *hda19* and *jmj14*, mutants adds to the evidence that their regulation is dependent on the coordinated action of many epigenetic complexes, as has been demonstrated for other plant defense genes [9].

Although no reports of epigenetic regulation in viral infection contexts for all 12 selected genes have been identified, evidence of epigenetic regulation in other contexts has been found for two POLV and one JMJ14 targets. *MTO 1 RESPONDING DOWN 1* (*MRD1*), a gene involved in SA biogenesis, was initially found to be severely downregulated in plants over-accumulating soluble methionine [48] and later found to be repressed in flowers of plants lacking the H3K36 methyltransferase SDG4 [49]. MRD1 has also been shown to be induced in *poliv* but not in the *polv* line SALK029919 [50]. Induction of *MRD1* was observed in our study in the *polv* line SALK017795C using both transcriptome and RT-qPCR methods (Fig. 4 and Table S3). Since this gene is highly susceptible to acquiring epigenetic variations in natural *A. thaliana* populations or in inbred epigenetic mutant lines [51–54], it is possible that the observed differences in the two *polv* mutant lines are not due to mutations but rather to spontaneous changes in the chromatin status of the locus. The DNA demethylase REPRESSOR OF SILENCING1 (ROS1) was shown to be required for removing methylation marks partially dependent on DICER-LIKE 2/DICER-LIKE 3 from a TE located in the promoter of the immune gene *RLP43*, allowing its induction when challenged with pathogen-derived flagellin [40]. The observed induction of the gene in a *polv* mutant line confirms that RdDM is required for buffering its expression in uninfected WT plants (Fig. 4 and Table S3). Finally, the histone deacetylase HDA19 and the histone binding WD40-repeat protein MULTICOPY SUPRESSOR OF IRA1 have been shown to directly regulate the ABA receptor coding gene *PYL5*, a predicted JMJ14 target [55]. In our experiments, the requirement of HDA19 for PYL5 expression was confirmed and its induction in the *clf* mutant provided further evidence that the gene is regulated by H3K27 marks (Fig. 5).

Because memory genes are frequently associated with epigenetic regulation in repeated stress situations, a temporally separated multiple plant virus infection experiment was designed to investigate the possible roles of the identified POLV- and JMJ14-regulated genes in WT plants. To our knowledge, no studies have investigated whether and how viruses influence plant responses when they are separated in time (i.e., a plant is transiently infected with one virus and then the same plant is infected by another virus). The combined analysis of the experiments revealed that if plants were previously stimulated with TMV, TRV or TuMV, they responded more efficiently against TuMV infection than unstimulated plants (Fig. 6). Furthermore, the level of protection increased in plants stimulated with TuMV compared to plants stimulated with the other two viruses. The role of signaling virus-derived sRNAs (vsRNAs) could be one explanation for the observed results. Due to the short duration of viral propagation (1 day) and the lack of viral templates for amplification in non-inoculated leaves, low levels of vsRNAs are expected to be produced during the stimulation phase, if any. These sRNA species, on the other hand, may have a minor role in directing TuMV silencing or, if host RNA targets are identified, in mounting appropriate epigenetic responses.

Transcriptomes from TuMV-stimulated, -unstimulated and controls were obtained to identify relevant memory genes associated with the virus stimulation effect. Most stress-related DEGs appeared to be downregulated in stimulated plants, reflecting the difference in viral titers between the two conditions at the sampled time point (Fig. 7c). However, in the induced set of genes, GO categories related to gene silencing and RdDM were enriched, indicating that epigenetic regulation may play a role in the observed virus induced resistance (Fig. 7c). It is reasonable to believe that only a small proportion of the DEGs between the two conditions are epigenetically regulated, and that some of these may be influenced by POLV and/or JMJ14. We found several stress-related genes that were deregulated in stimulated vs. unstimulated WT plants and were also present in the dataset of epigenetic protein candidates (Fig. 8a and Table S5). Six selected genes were quantified using RT-qPCR and were shown to be dynamically regulated during the infection process (Fig. 8c). Four of the examined genes (*CDC48B*, *LURP-LIKE1*, *RLP43*, and *RPW8*) did not exhibit the typical patterns of stronger and/or quicker expression usually seen in the induced resistance mechanism after being challenged with TuMV. At 8 dpi, these four genes were repressed in stimulated plants compared to unstimulated ones (Fig. 8c). This pattern of expression could be explained by the fact that, at this time point, virus-stimulated plants still had very low viral loads, comparable to those observed in unstimulated plants at 4 dpi. Typical priming behavior for these genes, if it occurred at all, could have occurred before 4 dpi or between 4 and 8 dpi and was overlooked in our study. However, the analysis was successful in identifying genes that were possibly primed for long-term expression after the challenge step, such as *PP2-A5* and *PYL5* (Fig. 8c). In total, 158 genes showed similar robustly increased induction (log_2_ fold change > 2) in low virus titer stimulated samples compared to the high virus titer unstimulated ones, despite the fact that the majority of them were not on the lists of POLV- or JMJ14-regulated ones (Table S4). This set contains several stress-related genes that could be direct or indirect targets of other epigenetics pathways. A detailed examination of the occupancy of epigenetic marks and proteins around the identified targets in virus-stimulated and unstimulated infected plants will be a useful tool for investigating the role of chromatin regulation in fine-tuning the expression of defense genes in viral infection contexts in plants.

## Methods

### Plant genotypes and growth conditions

*A. thaliana* (L.) Heynh of the Col-0 accession were cultivated under long day conditions, with 16 h of light (LED tubes at PAR 90–100 µmol m^−2^ s^−1^) at 24 °C and 8 h of darkness at 20 °C, in a mixture of 50% Kekkila substrate, 25% grade 3 vermiculite and 25% 3–6 mm perlite. Pest management was performed by the introduction of *Stratiolaelaps scimitus* and *Steinernema feltiae* (Koppert Corporation).

The *polv* (SALK 017795 C), *jmj14* (SALK 135,712 C), *atx1* (SALK_140755), *clf* (SALK_021003), *hda19* (SALK_139445), *kyp* (SALK_041474), *ref6* (SALK_001018C) and *sdg8* (SALK_026442) mutant genotypes were acquired from the Nottingham Arabidopsis Stock Centre. T-DNA genotyping was performed utilizing the LB-1.3 as the left-border primer, according to previously published procedures [56]. The gene-specific oligonucleotides used for genotyping are listed in Table S6. Using a 200 mM TRIS-HCL buffer (pH 7.5) containing 0.5% SDS, 250 mM NaCl, and 25 mM EDTA, DNA was extracted from small leaves and precipitated with isopropanol. PCR reactions were conducted using Thermo Scientific’s DreamTaq Green PCR Master Mix with the recommended conditions.

### Infection experiments

TuMV isolate YC5 (GenBank, AF530055.2) from calla lily plants [57] was utilized in all infection experiments. Infections were carried out by combining 0.1 g of ground-up infected sap tissue with 1 mL of 50 mM phosphate buffer (pH 7) containing 3% polyethylene glycol and 10% Carborundum, and then applying 5 µL of the mixture to two leaves of each plant. The same buffer solution was used for mock inoculations, but without the viral sap.

For the time-course experiments, mutant and WT control plants were inoculated (mock or TuMV) three weeks after germination, and non-inoculated leaves were harvested from separate batches of plants 4 and 7 dpi. Individual samples were taken, and each plant was allowed to continue developing in order to monitor symptoms. For each condition, batches of 12 plants (mock or visually confirmed infected plants) were pooled. Three biological replicates, each containing 12 plants, were obtained.

During the repeated virus infection experiments on WT plants, a total of 88 three-week-old plants were stimulated with TRV, TMV, ROS-fused TuMV, mock-inoculated as described above. A batch of 88 plants was also left without any treatments during the stimulation step to check if the abrasives used in the inoculation could by itself produce changed response to subsequent TuMV infection. To prevent the spread of the stimulating virus, inoculated leaves were removed 26–28 h after inoculation (Fig. S5), and 36 h after stimulation, plants from each condition were either challenged with TuMV (78 plants) or mock-inoculated (10 plants). From 5 to 20 days after TuMV challenge, symptoms were rated using the following scale: 0) no symptoms; (1) at least one leaf or stem with mild symptoms; (2) at least two leaves with clear yellowing; (3) all rosette leaves with clear yellowing; (4) all leaves with strong symptoms. At 4 and 8 dpi, random samples of nine infected and three control plants were taken from each condition for virus quantification and transcriptome analysis. The experiment was repeated six times and, in all cases, the full factorial setup was used.

### Transcriptome preparation and analysis

Total RNA was extracted with the GeneJET Plant RNA Purification Mini Kit (Thermo Scientific) and sent to the Novogene Europe for library preparation and sequencing. Messenger RNA was purified from total RNA using poly-T oligo-attached magnetic beads before library preparation. A directional library protocol was used for the mutant experiments. The library was checked with Qubit and real-time PCR for quantification and Bioanalyzer for size distribution detection. Quantified libraries were pooled and sequenced on the Illumina NovaSeq PE150 platform (minimum 6 Gb raw data per sample). For the mutant experiments, 48 libraries were sequenced from a combination of four genotypes (WT1, WT2, *polv*, *jmj14*), two conditions (mock, infected), two time-points (4 and 7 dpi), and three biological replicates (each with a pool of 12 plants). A total of 22 libraries were sequenced for the repeated virus infections in WT plants, with three conditions (mock, unstimulated, stimulated), one time-point (8 dpi), three individual plants for mock samples, four individual plants for unstimulated and stimulated conditions and two experimental set replicates.

The quality of the libraries was checked with FastQC v0.11.9 (https://github.com/s-andrews/FastQC) and trimmed with TrimGalore v0.6.6 (https://github.com/FelixKrueger/TrimGalore), using cutadapt v3.5 with Python 3.10.6 [58]. Ten bases from the 5’ end of reads 1 and 2 were removed before mapping with HiSat2 v2.2.1 [59] to the ENSEMBL release 51 of the *A. thaliana* TAIR10 genome assembly. Resulting SAM files were BAM-converted, sorted, indexed and analyzed with SAMtools v1.15.1 [60]. Read counting in features was done with htseq-count v0.11.1 [61], using The Arabidopsis Reference Transcript Dataset (AtRTD2) [62] as input annotation file. The count parameter -s was set to “reverse” for mutant libraries and “no” for repeated virus infection ones. Differential expression analysis was done with DESeq2 v1.36 [63] in R version 4.2.2, considering only genes having a total of at least 10 reads for each pairwise comparison.

### ChIP-seq analysis and integration

Data from previously reported ChIP-seq experiments was obtained from the NCBI GEO database [35, 64–68]. Libraries were prefetched and extracted with the SRA tool v2.11.2 [69] and checked, trimmed, mapped, and arranged as described before for transcriptome data (Table S2). Peak calling was performed using the normR R package [70]. As normR does not support biological replicates, each dataset was individually examined. Reads were counted in 250 bp tilling windows for H3K4m2, H3K4m3, and *NRPE1*; in 1 kb tilling windows for *JMJ14*, *NRPD1*, and 2 kb tilling windows for *DMS3*, *DRD1*, *RDM1*, and H3K9m2. When biological replicates were available (H3K4m2, H3K4m3, H3K9m2, and *NRPD1*), only peaks with *q*-values below 0.01 and shared by both were retained. Peaks with a *q*-value of zero were selected when just a single replication was available (*DMS3*, *DRD1*, *JMJ14*, *NRPE1*, and *RDM1*). The peaks from *DMS3*, *DRD1*, *NRPD1*, *NRPE1*, and *RDM1* were merged since they are all involved in the RdDM pathway. The subsetByOverlaps function of the GenomicRanges R package v1.48.0 [71] was used to obtain overlaps between DEGs and ChIP-seq peaks. This function was also used to obtain the metaplot with the percentage of TEs around the TSS of candidate direct regulated genes. The complete 6 kb region was segmented into 100 windows of 60 bp and overlapped with *A. thaliana* TE annotations [72]. Enrichment metaplots of H3K4m2/m3, H3K9m2, and *JMJ14* were computed using the R package genomation v1.28 [73], with the parameters weight.col and is.noCovNA set to “enrichment” and “TRUE”, respectively.

### WGBS-seq analysis

The NCBI GEO database was used to retrieve data from previously reported WGBS-seq experiments [74]. Libraries were prefetched, extracted, checked and trimmed as previously described for ChIP-seq data. Reads were mapped and deduplicated with the Bismark tool v0.23.0 [75] and the ENSEMBL release 51 of the TAIR10 genome assembly. SAMtools v1.15.1 was used for BAM sorting and indexing. Methylation calls were performed with Bismark in the “comprehensive” mode (information from the four strands were pooled). Methylation levels near the transcription start site (TSS) of direct regulated candidate genes were calculated using the genomation R package with 8 bp windows and the parameters weight.col and is.noCovNA set to “perc” and “TRUE,” respectively.

### Gene set functional characterization

Functional enrichment GO characterization was done with the R package clusterProfiler v4.4.4 [76] using the over representation analysis method. The R biomartr package v1.0.2 [77] was used for *A. thaliana* functional annotation retrieval. The enrichGO function was called with the following parameters: OrgDb = org.At.tair.db; keyType = “ENTREZID”; ont = “BP”; pAdjustMethod = “BH”; qvalueCutoff = 0.05; readable = TRUE; pool = FALSE. Ontologies were reduced with clusterProfiler’s simplify function and visual inspection in order to decrease redundancies. Genes were also classified based on MapMan bins of the X4 Araport11 R1.0 mapping file downloaded from the MapMan store website (https://mapman.gabipd.org/mapmanstore) [78] and with the Panther database 17.0 [79].

### RT-qPCRs

For RT-qPCRs, total RNAs treated with Turbo DNAse (ThermoFisher) were amplified in a 10 µL reaction with the PCRBIO 1-Step Go RT-PCR Kit (PCR Biosystems Ltd). Amplifications were performed on a StepOnePlus machine (Applied Biosystems) under the following cycling conditions: one cycle of retrotranscription at 45 °C for 10 min; one denaturing cycle of 95 °C for 2 min and 40 cycles of 95 °C for 5 s and 60 °C for 30 s; and a melting curve from 60 to 95 °C with 0.3 °C step increases. Using the Miner application [80], reaction efficiencies and the *C*_*T*_ values were calculated based on raw fluorescence. The HTqPCR R package v1.50.0 [81] was used to quantify transcripts with the comparative ΔΔ*C*_*T*_ method. As endogenous references, the previously identified *A. thaliana* stable genes *SAND* and *PP2A* were utilized [82]. The employed oligonucleotides are described in Table S6. The R package rstatix v0.7.0 (https://github.com/kassambara/rstatix) was used for carrying out Bonferroni-corrected pairwise *t* tests.

### Supplementary Information

**Supplementary Material 1.**

**Supplementary Material 2.**

**Supplementary Material 3.**

**Supplementary Material 4.**

**Supplementary Material 5.**

**Supplementary Material 6.**

**Supplementary Material 7.**

**Supplementary Material 8.**

**Supplementary Material 9.**

**Supplementary Material 10.**

**Supplementary Material 11.**

**Supplementary Material 12.**

## Data Availability

The data that support the findings of this study are publically available in the SRA database and can be viewed at the following URL: https://www.ncbi.nlm.nih.gov/bioproject/PRJNA973964.

## Abbreviations

ABA: Abscisic acid
ASCO: ALTERNATIVE SPLICING COMPETITOR
ATX1: ARABIDOPSIS TRITHORAX 1
CDC48B: CELL DIVISION CYCLE 48B
ChIP-Seq: Chromatin immunoprecipitation followed by next generation sequencing
CLF: CURLY LEAF
CNGC19: CYCLIC NUCLEOTIDE GATED CHANNEL 19
CSA1: CONSTITUTIVE SHADE-AVOIDANCE1
DEGs: Differentially expressed genes
DMS3: DEFECTIVE IN MERISTEM SILENCING 3
dpi: Days post-inoculation
DRD1: DEFECTIVE IN RNA-DIRECTED DNA METHYLATION 1
ETI: Effector-triggered immunity
GO: Gene ontology
HDA19: HISTONE DEACETYLASE 19
HR: Hypersensitive response
JA: Jasmonic acid
JMJ14: JUMONJI14
kb: Kilobases
KYP: KRYPTONITE
LURP: LATE UPREGULATED IN RESPONSE TO HYALOPERONOSPORA PARASITICA
mC: Methylcytosine
MRD1: MTO 1 RESPONDING DOWN 1
NAC003: NAC DOMAIN CONTAINING PROTEIN 3
NLR: Nucleotide-binding oligomerization domain (NOD)-like resistance
NOD: Nucleotide-binding oligomerization domain
PCA: Principal component analysis
PCSD: Plant Chromatin State Database
POLIV: RNA Polymerase IV
POLV: RNA Polymerase V
PP2-A5: PHLOEM PROTEIN 2 A5
PTI: Pattern-triggered immunity
PYL5: PYRABACTIN RESISTANCE 1-LIKE 5
REF6: RELATIVE OF EARLY FLOWERING 6
RdDM: RNA-directed DNA methylation
RDM1: RNA-DIRECTED DNA METHYLATION 1
RGL3: RGA-LIKE PROTEIN 3
RLP43: RECEPTOR LIKE PROTEIN 43
ROS1: REPRESSOR OF SILENCING1
RPW8: RESISTANCE TO POWDERY MILDEW 8
PRC2: Polycomb repressive complex 2
RT-qPCR: Reverse transcription quantitative polymerase chain reaction
SA: Salicylic acid
SDG8: SET DOMAIN GROUP 8
sRNAs: Small RNAs
TE: Transposable element
TF: Transcriptional factors
TMV: Tobacco mosaic virus
TRV: Tobacco rattle virus
TSS: Transcription start sites
TuMV: Turnip mosaic virus
WGBS-seq: Whole genome bisulfite sequencing
WT: Wild-type
