# Supplementary figures and images for: Identification of epigenetically regulated genes involved in plant-virus interaction and their role in virus-triggered induced resistance
Source: BMC Plant Biol. 2024 Mar 5;24:172. doi: 10.1186/s12870-024-04866-3 (PMC10913459; doi:10.1186/s12870-024-04866-3)

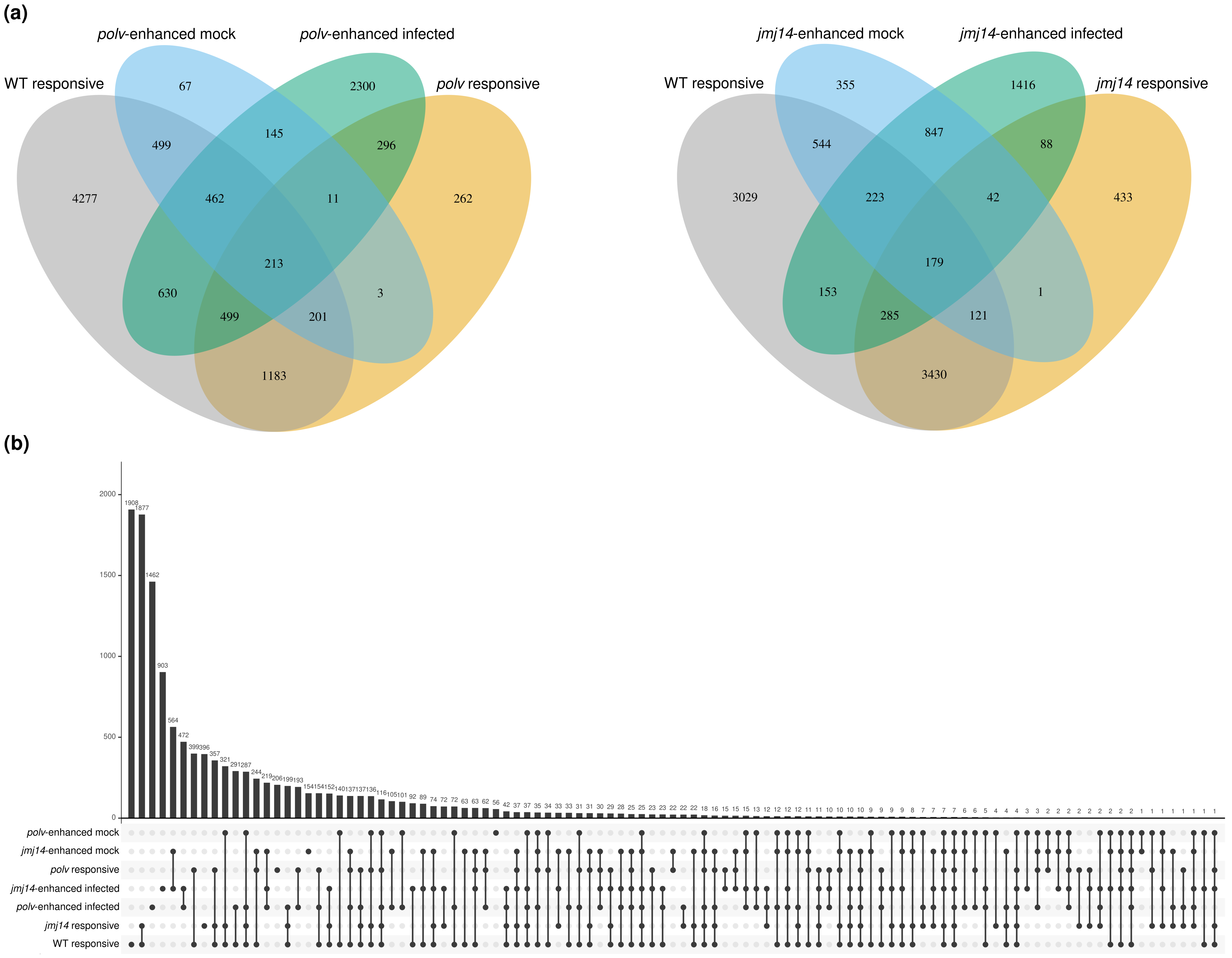

Supplement: Supplementary file 1 — Supplementary Material 1. [file 12870_2024_4866_MOESM1_ESM.png]

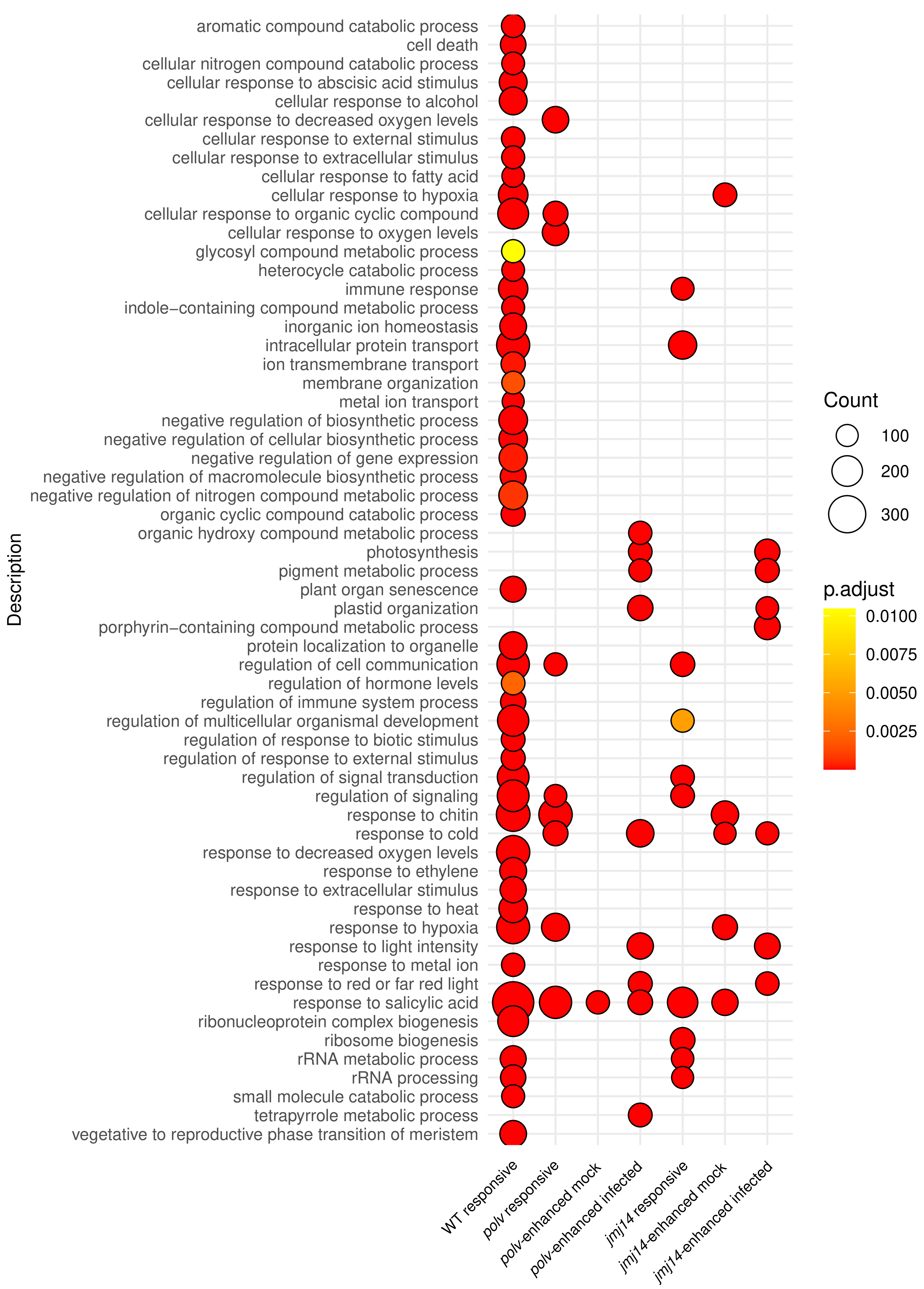

Supplement: Supplementary file 2 — Supplementary Material 2. [file 12870_2024_4866_MOESM2_ESM.png]

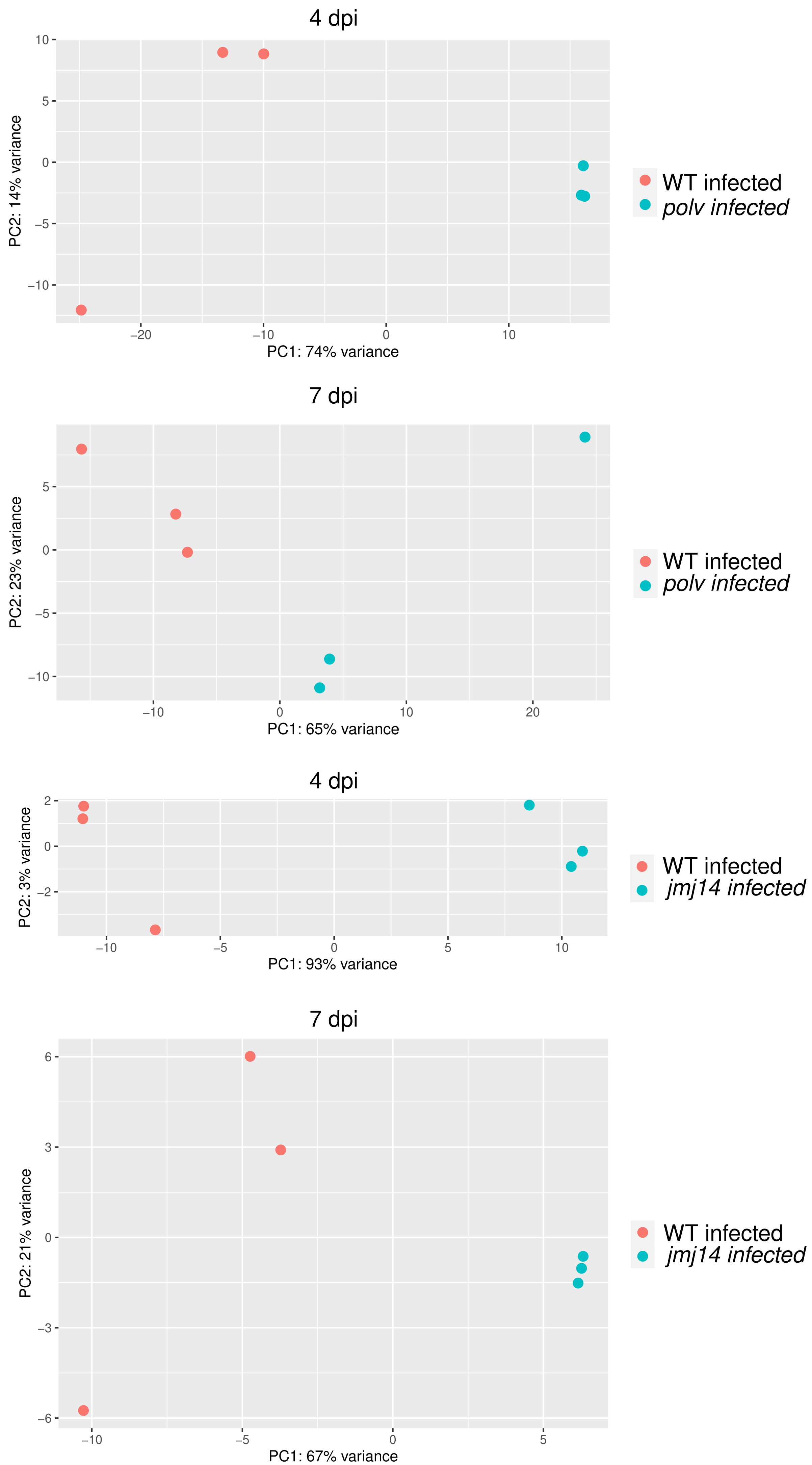

Supplement: Supplementary file 3 — Supplementary Material 3. [file 12870_2024_4866_MOESM3_ESM.png]

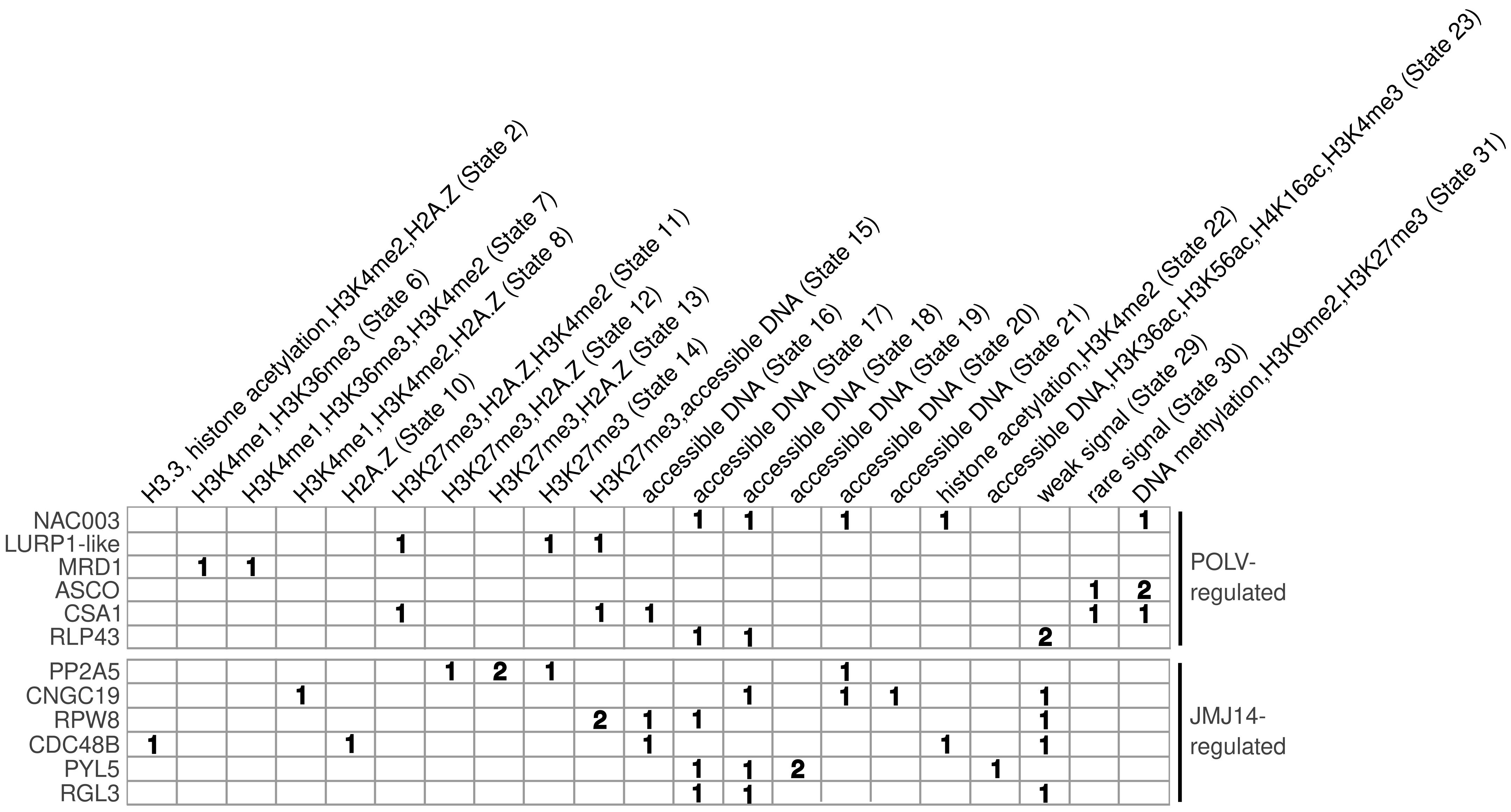

Supplement: Supplementary file 4 — Supplementary Material 4. [file 12870_2024_4866_MOESM4_ESM.png]

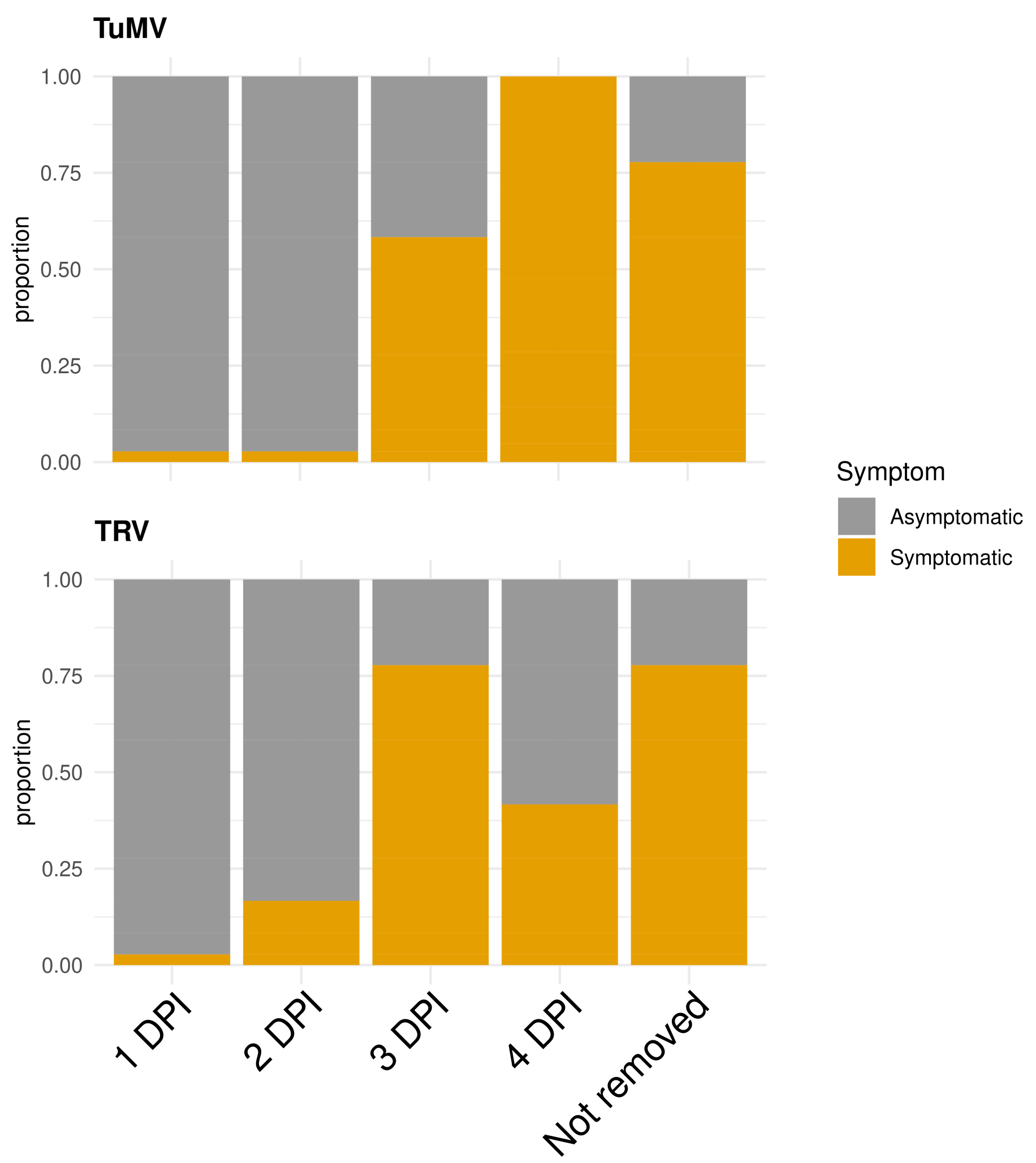

Supplement: Supplementary file 5 — Supplementary Material 5. [file 12870_2024_4866_MOESM5_ESM.png]

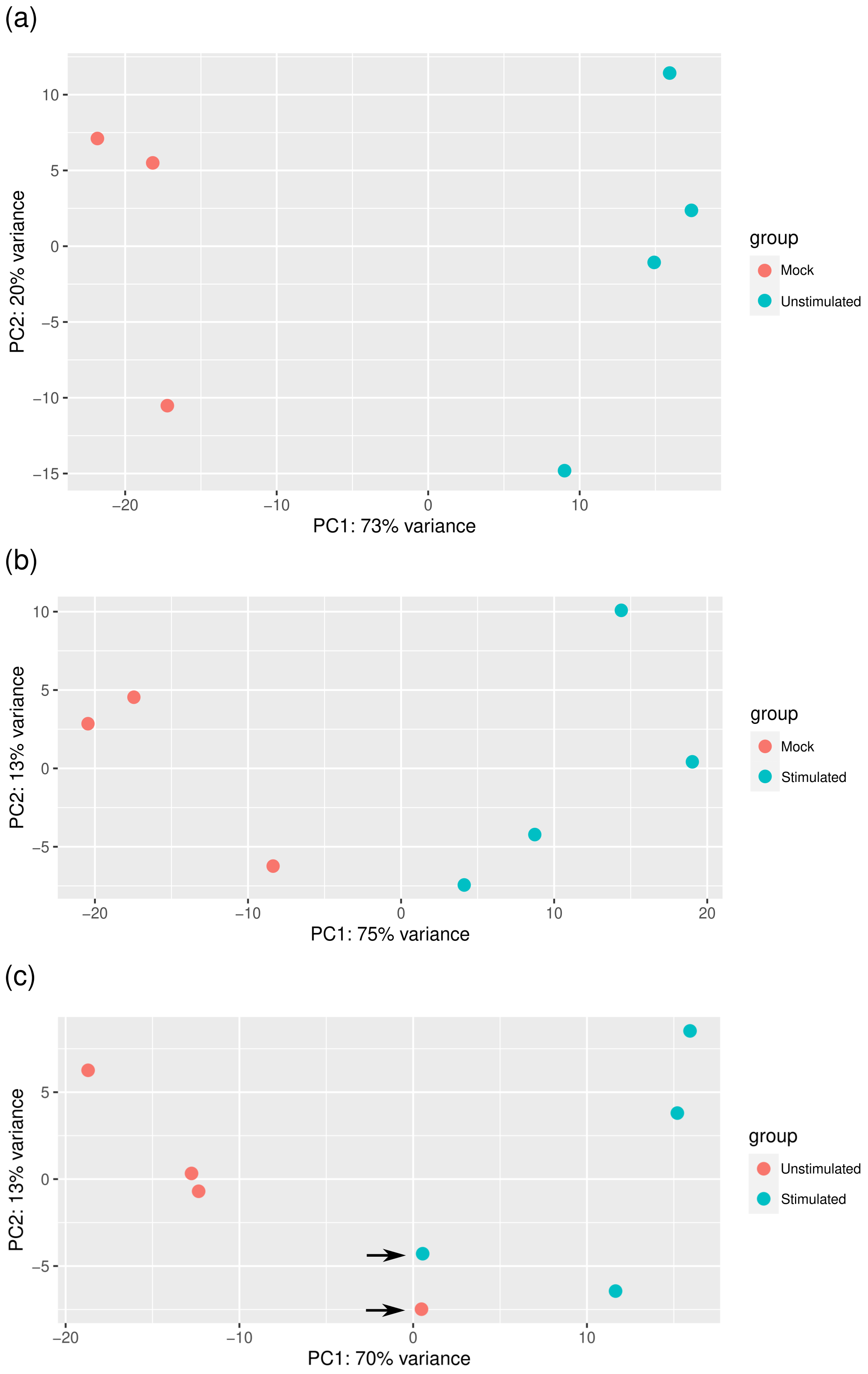

Supplement: Supplementary file 6 — Supplementary Material 6. [file 12870_2024_4866_MOESM6_ESM.png]
